# Foraging, Farming or Shopping? A Decision Matrix Approach for Food Environment Assessments

**DOI:** 10.3390/ijerph22050711

**Published:** 2025-04-30

**Authors:** Lilly Zeitler, Suwichan Phatthanaphraiwan, Shauna Downs, Bronwen Powell

**Affiliations:** 1 Department of Geography, The Pennsylvania State University, University Park, PA 16802, USA; bxp15@psu.edu; 2 School of Liberal Arts, Mae Fah Luang University, Chiang Rai 57100, Thailand; suwichan.pha@mfu.ac.th; 3 Department of Health Behavior, Society and Policy, Newark, NJ 07102, USA; sd1081@sph.rutgers.edu; 4 African Studies Program, The Pennsylvania State University, University Park, PA 16802, USA

**Keywords:** multi-criteria decision analysis, food choice, wild foods, food sharing, food quality, decision-making, Indigenous, low-and-middle-income countries, participatory mapping, mixed methods

## Abstract

Food environments (the interfaces between consumers and the broader food system) shape dietary change and associated health outcomes. Characteristics of food environments (e.g., availability, accessibility, affordability, convenience, desirability) can influence consumer decision-making around food acquisition in different types of food environments (e.g., informal and formal markets, wild and cultivated natural environments). With the novel decision matrix approach presented in this paper, we aimed to develop a simple and rapid tool for collecting perceived evaluations and preferences of different types and characteristics of food environments. The decision matrix results were triangulated using a mixed methodology of geolocated participant observation, participatory mapping, market price comparisons and qualitative interviews. The decision matrix results were compared to the reported use of different food environment types in an Indigenous Pgaz K’Nyau community in Northern Thailand. Despite an ongoing food environment transition, participants preferred natural food environments and ranked market environments most poorly, largely reflecting actual food environment use. Interviewees stressed the importance of flavor and food safety, citing concerns over agrochemical contamination of market foods. The proposed decision matrix and mixed methods approach provides a rapid data collection method that can be used by food environment researchers and public health practitioners to assess food environment preferences and perceptions that influence decision-making in food environment transitions in low- and middle-income countries.

## 1. Introduction

From small decisions (e.g., choosing a snack) to large decisions (e.g., starting a business, moving abroad, etc.), humans constantly engage in decision-making. Interest in the processes behind individual decision-making has led to the development of decision science and analysis [1,2,3]. Multi-criteria decision analysis is used to evaluate different options by a range of criteria, which can be arranged in a decision matrix to quantify the best possible option (the maximization case) [4,5].

Decision science and analysis is “polytheoretical” and involves many disciplines [6]. Multi-criteria decision analysis has been applied to a range of fields, including health care [7], agriculture [8], energy infrastructure [9], nutrition-sensitive agriculture [10], nature conservation [11], environmental studies [12,13], and the agri-food sector [14,15]. In the food sector, multi-criteria decision analysis has been used to enhance sustainability outcomes, promote participatory approaches and develop key indicators [14]. Improving environmental impacts [16], food safety [17,18,19,20], food engineering [21], food waste treatment [22,23,24,25,26,27,28,29,30,31], marketing of local food products [32], supply chain optimization [33] and sensory evaluations of food products [34,35,36] are some of the multi-criteria decision analysis applications in food systems research.

Multi-criteria decision analysis in food environment research is still limited. GIS-based multi-criteria decision analysis has been utilized to study distance and transportation to different retailers in food deserts [37]. Recent innovative work uses decision trees to analyze food environment decision-making [38]. Multi-criteria decision analysis has not yet been brought into conversation with theoretical developments on different types [39] and characteristics [40] of food environments, presenting an exciting opportunity for new avenues of multi-criteria decision analysis in food environment research.

Food choice in food environments involves multiple decisions from food acquisition to preparation, storage and consumption [6,41,42]. The average individual is estimated to make ~220 food-related decisions per day [43]. Some of these decisions are conscious; however, many are subconscious; they are situational, complex and multifaceted [6]. While decisions around food consumption are often impulsive, emotional and subconscious (*implicit decision-making*) [43,44], value negotiations for food acquisition tend to be more conscious (*explicit decision-making*) [43]. In addition to broader cultural and social–ecological contexts [6,39], food acquisition decision-making processes for choosing one type of food environment over another, such as a supermarket (a formal market environment) over a home garden (a natural food environment), are guided by decision-making factors—known interchangeably as dimensions, aspects, attributes or characteristics of food environments.

Numerous methods have been employed to measure the food environment characteristics that influence decision-making [38,40,45]. Food environment measurement tools and methods for high-income countries are generally better developed than those for low- and-middle-income countries (LMICs) [46,47]. Some of the food environment assessment frameworks developed for high-income countries have been adapted to LMICs, such as for African contexts [48]. Recent efforts have greatly enhanced informal market assessments in LMICs [49,50,51,52,53]. However, approaches that include all types of food environments are lacking [45]. Farrell and colleagues further note that “there remains a need for conceptual advancement in the area of measures and metrics to **measure multiple characteristics** within **evolving food environments** in order to identify opportunities for change” [24] (emphasis added). Our proposed mixed methods and decision matrix approach responds to this call to further develop methods for assessing multiple characteristics in food environment transitions.

Food environments are not static or fixed but rather are dynamic and evolve over time. Downs et al. [39] propose the term “food environment transitions” to denote both short-term seasonal and long-term gradual shifts from nature- to market-dependent societies. Changing food acquisition practices from predominately natural to market food environments is proposed as a key driver of dietary change with associated health outcomes [39,54]. Indigenous communities are especially impacted by food environment transitions [55,56,57,58]. Increased market access has been associated with lower dietary diversity and increased sugar and fat intake in former hunter–gatherer societies [58]. Substituting traditional foods with market foods has been associated with poorer nutrition and obesity outcomes in Indigenous communities [57]. Better understanding of changing food environment preferences within food environment transitions, especially in Indigenous and minoritized contexts, is needed to understand broader trends of market integration, dietary change and their implications for public health.

The purpose of this paper is to develop a mixed methods approach for assessing and measuring individuals’ perceptions of food environment characteristics for different types of food environments to help explain why individuals may prefer and choose one type of food environment over another, and how these preferences might change in food environment transitions. Our approach focuses on Indigenous and rural food environments undergoing transition in LMICs and is designed to provide insights into individuals’ perceptions of food environment characteristics that can help explain consumer–food environment interactions, food-related behaviors, food choices and potential dietary outcomes. We apply our mixed methods approach for developing food environment evaluations in a case study of Indigenous Pgaz K’Nyau food environment transitions in Chiang Mai Province, Northern Thailand.

## 2. Materials and Methods

### 2.1. The Decision Matrix Approach

The mixed methods approach includes a simple but effective food environment preference ranking focus group protocol that can be converted into a multi-criteria decision matrix. We present a (<1 h) focus group protocol that can be used to develop a multi-criteria decision matrix to better understand food environment decision-making in diverse contexts, including in Indigenous and rural food environment transitions in LMICs (see the focus group protocol in the SI).

According to multiple criteria decision-making analysis, multi-attribute utility theory and preference theory [59], decision-making processes involve evaluating different options or prospects based on various criteria. For instance, when purchasing a new car, multiple options (car makes and models) may be evaluated based on different criteria (safety, appearance, gas mileage, comfort, status, etc.). In the realm of food environments, individuals may choose between different options or types of food environments (e.g., a local bodega, a fast-food outlet, a supermarket, a home garden, a forest, etc.) based on different evaluation criteria (often referred to as food environment characteristics, aspects, dimensions or attributes), including availability, affordability and price, accessibility, convenience, desirability, vendor and product properties, etc. [40]. Multi-criteria decision analysis methods quantify the best or worst option based on multiple decision-making criteria.

Multi-criteria decision analysis is an umbrella term encompassing many different models and methods (both quantitative and qualitative) used to study decision-making [60,61]. We propose using the simplest and most well-known multi-criteria decision analysis (MCDA) method: the weighted sum model. The weighted sum equation is the sum of weighted criteria (*C_j_ W_j_*) for each option (*i*):

$$A_{i}WSM\;score=\sum_{j}^{n}C_{j}\;W_{j}\;,for\;i=1,\;2,\;3,\ldots m.$$

Out of m alternative options (*A_i_* for *i* = 1, 2, 3, … *m*), the best option based on *n* decision-making criteria (*C_j_* for *j* = 1, 2, 3, … *n*) that are weighted (*W_j_* for *j* = 1, 2, 3, … *n*) would have the highest weighted sum model score (the maximization case). The worst option would have the lowest weighted sum model score. According to preference and prospect theory, individuals are most likely to select the best option available, based on the premise that humans are utility maximizers and seek to minimize risk, unless losses are guaranteed [62,63,64]. A decision matrix can therefore help evaluate the logic behind why one option may be chosen over another. Weighting different criteria can also help explain decision-making amidst multiple trade-offs (which may be referred to as value negotiations in the food choice literature) [6]. Table A1 in Appendix A illustrates an adapted weighted sum model calculation for food environment assessments, drawing from Herforth and Ahmed’s [65], Turner et al.’s [40] and Downs et al.’s [39] food environment frameworks.

The primary benefit of the decision matrix and weighted sum model approach is that it is flexible and can expand or shrink as new options and criteria are added or removed. In the ever-evolving world of food environment frameworks, this presents a high-utility advantage. Since publication of Downs et al.’s [39] food environment typology, two new types of food environments have already been proposed: kin and community [66] and wild–cultivated [67] types of food environments. Following Herforth and Ahmed’s [65] four initial food environment characteristics (availability, affordability, convenience and desirability), Turner et al. [40] have expanded these to eight dimensions (internal domains: accessibility, affordability, convenience, desirability; external domains: availability, prices, vendor and product properties, marketing and regulations), while Farrell et al. [45] considered nine characteristics (healthiness, availability, affordability, accessibility, convenience, environmental sustainability, food security, desirability and food safety). A flexible approach is needed to accommodate the dynamism of food environment conceptual developments. We present the decision matrix approach as a simple method that can merge two aspects of food environment conceptualizations, “types of food environments” and “food environment characteristics”, into a single model. Importantly, the model is easy, fast and inexpensive to apply in food environment assessments, including in LMICs.

### 2.2. The Mixed Methods Approach

The proposed mixed methods are designed to triangulate the decision matrix results and facilitate a comprehensive assessment of individuals’ perceptions of different types of food environments based on their characteristics. The mixed methods approach includes focus groups, interviews, participatory mapping, geolocated participant observation and market price comparisons (see Table 1). While some approaches aim to capture objective measures of food environments, we focus on subjective perceptions, as ultimately, food environment decision-making will be guided by individuals’ perceptions [41]. Subjective evaluations of characteristics across different types of food environments were assessed using a preference ranking protocol in focus groups to produce the decision matrix. Qualitative interviews were conducted to contextualize and understand the reasons behind the focus group preference ranking results in greater depth. Objective food environment measures were collected for two food environment characteristics: price and access. Price data were collected using market price comparisons. Access data (with distance as a proxy measure) were collected using geolocated participant observation and participatory mapping in focus groups. The comprehensive mixed methods approach is designed to triangulate the decision matrix results.

**Figure 1:**
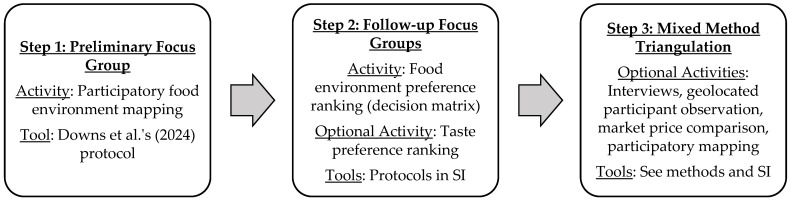
A suggested workflow diagram for implementing the proposed methodological approach. The modular design allows for activities in step 2 and 3 to be added or removed, depending on project objectives [68].

Figure 1 provides a suggested workflow diagram for the proposed methods. The modular research design allows for activities to be added or removed based on research project objectives. Studies that only aim to develop or include a decision matrix can simply follow steps 1 and 2 and exclude step 3. Mixed methods for triangulation can be selected from the diverse range of “optional” activities in step 3, as not all activities may be necessary for all studies.

### 2.3. Practical Case Study Application

#### 2.3.1. Case Study Site

We apply the decision matrix and mixed methods approach to a case study of Indigenous Pgaz K’Nyau food environments in San Din Daeng village, Baan Luang subdistrict, Chom Thong district, Chiang Mai province—located approximately 80 km southwest of Chiang Mai. Indigenous Pgaz K’Nyau Peoples are an ethnic Karen subgroup and the largest ethnic minority in Thailand. Pgaz K’Nyau food environments are in the process of transitioning from natural food environments toward built market environments (Pattern 3 as per Downs et al.’s [39] food environment transition model). Recent and ongoing smallholder maize commercialization (<5 years) is likely triggering a “food environment transition” from agrobiodiverse subsistence fields toward monoculture cash crops with increasing market orientation. Infrastructure development projects (e.g., road and bridge construction) enhance market access, which in conjunction with increasing incomes from animal feed corn sales, spurs more frequent market interactions. The study site provides an opportunity to develop and apply methodologies that can assess community members’ perceptions of various characteristics of different food environment types in an ongoing Indigenous and rural food environment transition.

#### 2.3.2. Focus Groups

A total of sixteen women, representing a range of age groups and nearly half of the households in San Din Daeng village, were invited to participate in four focus groups. A local Pgaz K’Nyau research assistant recruited participants aiming to include diverse religions (Christian and Buddhist) and ages (24–65 years old) for a sample representing diversity in the community. Women tend to be the primary household food decision-makers [69,70,71], so only female participants were included. The total sample size of 16 women provides coverage of nearly half the households in San Din Daeng (43%), offering a more representative sample than a single focus group of 8 women. Each focus group included 7–8 women, because this was a group size well suited for engagement and participation, and provided each woman with an opportunity to speak within the allocated time frame. The focus group protocols were developed by the lead author with inputs from all the co-authors (see the SI for focus group protocols). All the focus groups were facilitated by the lead author, who speaks fluent Thai. Participants were compensated with 300 THB (~USD 8.80)—the equivalent of a daily local wage.

**Figure 2:**
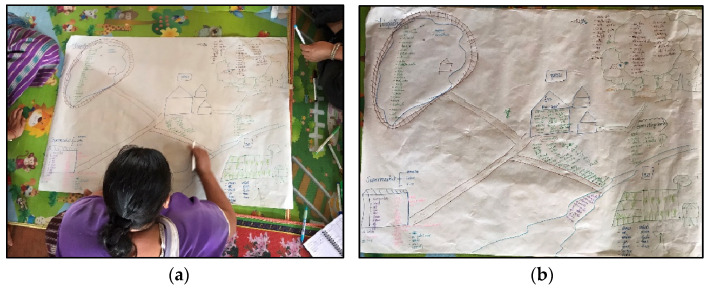
Hand-drawn community food environment map developed using the Downs et al. [68] protocol: (**a**) a focus group participant adding to the community food environment map, and (**b**) the final hand-drawn community food environment map.

The focus group in step 1 (see Figure 1) followed Downs et al.’s [68] participatory community food environment mapping protocol to develop an emic classification of different types of food environments (n = 7, see Figure 2 and Table 2). The local types of food environments identified in the step 1 focus group were then used to pilot the step 2 focus group protocols for a simplified food environment and taste preference ranking activity that was used to produce the decision matrix (n = 8; see the Supplementary File S1: Focus Group Protocol and Data Collection Sheets; see Table A2 in Appendix A for the pilot focus group results). Following lengthy discussions with the pilot focus group participants about how to rank food safety, flavor, satiety, healthiness, environmental sustainability and cultural appropriateness all under a single characteristic of “desirability,” we decided to develop an expanded food environment preference ranking focus group protocol that disaggregated desirability into distinct characteristics. Both the simplified and expanded preference ranking focus group protocols can be found in the SI.

We conducted two focus groups in step 2 and included three activities: the expanded food environment preference ranking exercise, a taste preference ranking exercise and a participatory mapping activity (see step 2 in Figure 1; see the SI for the focus group protocols). The expanded food environment preference ranking protocol included five types of food environments (wild, wild–cultivated, informal market, formal market, and kin and community), ten food environment characteristics (availability, access, affordability, convenience, desirability, safety and freshness, healthiness, sustainability and cultural appropriateness) and three individual factors that influence desirability (taste, satiety, affect). Focus group participants ranked the food environment characteristics for each type of food environment on a Likert scale from 1 to 5 (not good at all, not good, average, good, very good) and weighted each characteristic on a Likert scale from 1 to 5 (not important at all, not important, average, important, very important). The ranked values for each characteristic and type of food environment were weighted and then averaged across all the participants as inputs for the final decision matrix. The average ranked values for all the characteristics were added together to generate the final average weighted sum score for each type of food environment. The two back-to-back focus groups with three activities each (food environment preference ranking, taste preference ranking and participatory mapping) took approximately 4 h, from 2 pm until 5:50 pm in the afternoon (n = 16).

#### 2.3.3. Participatory Mapping

We included an additional participatory mapping exercise in the focus groups, which encouraged community members to engage with the mapping process. Google Earth satellite images of the village and surrounding areas were printed on sheets of A4 paper. Focus group participants were asked to mark food acquisition locations and specify the foods acquired from each location. The participatory mapping focus group results were digitized using ArcGIS Pro software (version 3.1.0) to produce a community food environment map and to calculate the distances from the village to compare accessibility between different types of food environments.

#### 2.3.4. Geolocated Participant Observation

**Figure 4:**
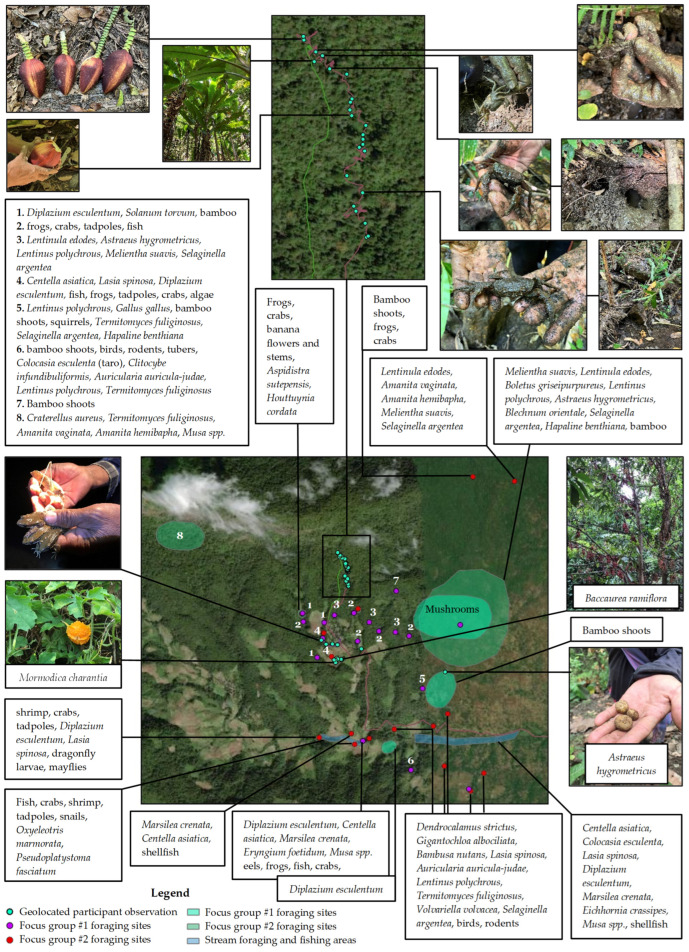
Community food environment map based on geolocated participant observation and focus group data.

The lead author conducted ongoing participant observation throughout the research project, including going on foraging outings with five community members over three days and geolocating their foraging locations using a Garmin eTrex 10 GPS device. Notes about the acquired foods were entered into the GPS device for each location. The lead author also accompanied community members on shopping trips and recorded their shopping locations in town. Travel times to foraging and shopping locations were recorded in field notes. The foraging and shopping GPX data were uploaded to ArcGIS Pro software. Distances of tracks to different food environments were calculated using the calculate geometry tool in ArcGIS Pro. Distances without track data were calculated using the distance measuring tool. For a georeferenced community food environment map based on participatory mapping focus groups and geolocated participant observation data, see Figure 4.

#### 2.3.5. Qualitative Interviews

Seven qualitative interviews were conducted with San Din Daeng residents on the characteristics of different types of food environments. Interviewees were purposively sampled to include women across a range of ages. The interview questions were designed to better understand individual decision-making, as well as community-level factors that influence the characteristics of local food environments. A local government health official, a village leader and a local health volunteer were interviewed for specialist opinions on food environment change in the community. Interviews were conducted in Thai language or in a mix of Thai and Pgaz K’Nyau language with a Pgaz K’Nyau–Thai translator. All the interviews were translated into English. The interview translations were coded deductively by the lead author for each food environment characteristic (availability, affordability, accessibility, convenience, desirability, food safety, freshness, sustainability, cultural appropriateness) and individual factor (taste, satiety, affect) for each type of food environment (wild, wild–cultivated, informal market, formal market, kin and community). The codebook was developed collaboratively by the lead author with inputs from the other authors. B.P. and the lead author reviewed the codes until consensus was reached. Interview excerpts were extracted and arranged in a table for each food environment characteristic by the type of food environment in Microsoft Word.

#### 2.3.6. Market Price Comparison

A market price comparison was conducted between a supermarket (Tesco Lotus) and a fresh market in Chom Thong town to compare formal and informal market prices by food group. Prices and photographs of comparable food items were collected on three days in three different seasons. For instance, if broccoli, morning glory and kale were available in both the supermarket and the fresh market, the prices would be recorded for each item at each location. The mean price for all the food items were then calculated for an aggregate average price for the “dark green leafy vegetable” food group. This process was repeated for all the food groups included in the Minimum Dietary Diversity for Women (MDD-W) metric. The prices were standardized into the cost per kilogram for comparison and converted to US dollars (USD) in Excel. The market price comparison data are available in the SI.

## 3. Results

### 3.1. Types of Food Environments

**Figure 3:**
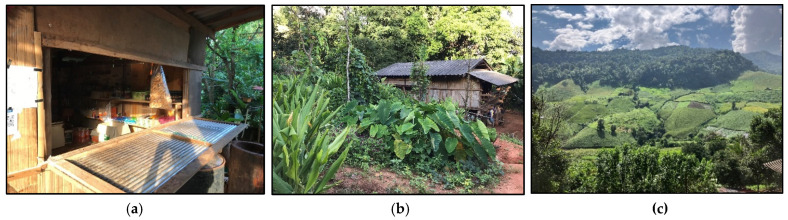
Examples of food environments in San Din Daeng: (**a**) an informal market village kiosk, (**b**) a home garden, and (**c**) rotational crop fields.

San Din Daeng residents acquired foods from diverse food environments (see Table 2 and Figure 3). Focus group participants first classified wild and cultivated types of natural food environments as two separate types of natural food environments. However, while discussing rice paddies, they noted the presence of aquatic wild species, such as fish and crabs, co-existing with cultivated rice. Focus group participants concluded that these environments were both wild and cultivated, which we have called a “wild–cultivated” type of food environment [67]. Other wild–cultivated food environments included swiddens, home gardens and agricultural streams with a mix of wild and cultivated elements (see Table 2). Purely cultivated environments without wild foods were rare. Informal market environments in the village included one village kiosk that sold sodas, sweets, and dried and canned goods. The markets in Chom Thong town included informal shops and fresh markets, and formal supermarkets and convenience stores. Though animal husbandry is practiced in forests, focus group participants considered forest environments “wild”. Food sharing with kin and community is a common everyday practice and can occur in homes, at roadsides and with friends and relatives in different villages.

**Table 2:** Emic food environment classifications by the San Din Daeng focus group participants.

Type of Food Environment	Examples of Places
Wild	Forest, forest streams
Wild–Cultivated	Rice paddies, rotational farms, home gardens, agricultural streams
Cultivated	Non-swidden monoculture fields without wild foods
Informal Markets	Village kiosks, fresh market, informal shops, roadside vendors
Formal Markets	Convenience stores (e.g., 7-Eleven), supermarkets (e.g., Tesco, Big C)
Kin and Community	Homes, paths and roads, fields and forests, different villages

### 3.2. Decision Matrix Preference Ranking of Food Environments Types

Natural food environments were preferred over built food environments for all food environment characteristics (see Table 3). Wild–cultivated food environments were the most preferred type of food environment and given the highest ranking of an average of 3.8 out of 5 (corresponding to “good”) overall (see Table 3). Wild–cultivated food environments were ranked highest for the characteristics of convenience, accessibility, availability and desirability, relative to other types of food environments. Wild food environments were ranked highly (weighted average of 3.7 out of 5), with the highest ranking for food safety, freshness, affordability, healthiness, sustainability, taste and satiety. Informal markets were ranked lower (weighted average of 2.2 out of 5): “not good” in terms of affordability and cultural appropriateness, and “average” in terms of convenience, accessibility, availability and desirability. Formal markets were ranked most poorly (weighted average of 1.4 out of 5) and “not good at all” overall.

Food environment preferences varied between age groups (see Table 4). Market environments were ranked most poorly across all age categories. Wild–cultivated and wild food environments were ranked most highly across all age groups; however, the oldest age category ranked them more favorably than younger participants. Kin and community food environments were ranked most favorably by the oldest age category and most poorly by the youngest age category. As people age, they become greater beneficiaries and recipients of food sharing, whereas younger individuals are often expected to share food with elders.

### 3.3. How Food Environment Characteristics Shape Preferences and Decision Making

The mixed method results largely support the results from the decision matrix analysis. Across different methodological approaches, participants expressed a strong preference for wild and wild–cultivated food environments due to their convenience, accessibility, affordability, availability and desirability. Our geolocated participant observation, participatory mapping and market price comparisons support the qualitative interview and focus group findings that foods from natural food environments are less expensive and generally more convenient, accessible and desirable. Themes emerging from the interview data included the different food environment characteristics, synergies between taste and food safety, and decision-making trade-offs. The interview data provided important context for interpreting the preference rankings in the decision matrix.

#### 3.3.1. Desirability and Food Properties: Taste and Food Safety

Food safety and taste were the food environment characteristics that interviewees discussed most frequently and at length. These were also the characteristics weighted most highly in the decision matrix (see Table 3). Multiple interviewees noted the synergies between flavor and food safety in wild–cultivated food environments. One interviewee summarized these synergies as: “*The main thing is growing and eating it ourselves. It tastes better*, *it’s safer*, *and if we don’t have money*, *we don’t have to worry*” (Interviewee in her 40s). Another interviewee concurred, emphasizing food safety and flavor: “*If you can grow it yourself then it’s safer*, *better*, *tastier. Down there [in markets in town]*, *there’s a lot of chemicals*” (Interviewee in her 30s). Another interviewee explained the close relationship between flavor and food safety: *“The taste is different, because it’s sweeter, more delicious, and more fragrant. When we buy from the market, we don’t know if they use chemicals … The [vegetables] they sell, especially the good-looking ones, are sprayed with insecticides, which makes them less tasty. Ours, on the other hand, are chemical-free, so it’s more reassuring to eat, and the taste is naturally better”* (Interviewee in her early 40s).

Another interviewee linked food safety to flavor by noting how agrochemicals change the texture, flavor and healthiness of market produce: *“The difference [between highland and lowland food] is huge, especially in taste. The taste has changed a lot. Down in the lowlands, it’s not delicious, not sweet, and it doesn’t have much flavor. The vitamins are gone. Sometimes, even with the same vegetables, the taste is different. When we make curry, you can really tell. If they don’t use fertilizer, you can tell right away. When we cook it [the vegetables], after about 10 to 15 min, it’s soft and done. But if they use fertilizer, it takes much longer to cook, and it still doesn’t get soft. You can really feel the difference. You can tell when they use pesticides or when they don’t”.*

People linked gustatory and olfactory sensory properties to the healthiness and affordability of highland rice as compared to jasmine rice from the market: “*The rice we grow ourselves is sweeter and more fragrant. When we buy rice from the market*, *it’s bland*, *and I feel like the nutrients are gone. Jasmine rice is expensive*, *but it doesn’t taste as good*, *and the vitamins are lost in the process of preservation*” (Interviewee in her early 40s).

Most interviewees expressed a strong preference for the flavors and textures of wild and wild–cultivated “mountain” foods from natural food environments over market foods, expressing some variation of “*The food from the mountains is most delicious*” (Interviewee in her late 30s). However, one younger interviewee noted novelty as a factor for enjoying market foods: “*I like market food*, *because I eat forest food a lot and can get tired of it. I like trying something new at the market*” (Interviewee in her early 20s). A preference for the flavors of stream-caught fish and self-raised animals over market-bought fish and meat was expressed by several interviewees: “*Fish caught from the stream is also tastier than fish bought in the city*” (Interviewee in her early 20s), and “*The meat from animals raised in the hills is more delicious*” (Interviewee in her early 40s). One interviewee compared both the flavor and texture of animals raised on the mountain in natural food environments to market varieties: “*The pork and chicken we raise are more delicious*, *such as black pork. Chicken from the city is bland and tough*, *but from the mountain, it is sweeter and softer. Mountain pork has more fat and is tastier*” (Interviewee in her early 20s). Interview data on the sensory properties of taste, but also fragrance and texture, supported the high taste preference ranking for wild and wild–cultivated types of food environments and the low rankings for built market types of food environments. Interviewees’ statements confirmed the focus group participants’ ranking of taste as “very good” in natural food environments and as “not good” in markets.

Taste was the food environment characteristic weighted most highly by focus group participants (see Table 3). Based on our taste preference ranking results, older participants preferred bitter flavors and disliked sweet, fatty and salty flavors (see Table 5). Younger participants preferred salty and fatty flavors the most. Focus group participants associated sweet and fatty flavors with built food environments in town. However, some participants noted that you can deep fry bananas from your home garden for similar flavors. Bitter flavors were associated with wild and wild–cultivated types of food environments. Bitter gourd, or *Momordica charantia*, was listed most often as an example of a bitter food. The intergenerational changing taste preferences appear to reflect increasing market use.

Interviewees shared food safety concerns over agrochemical contamination of market foods, disease, poor hygiene, and freshness (see Table 6). The primary food safety concern emphasized by all the interviewees across all age groups was agrochemical residues and contamination. Foods from market environments were perceived as unsafe and “likely dangerous” due to agrochemicals. By contrast, home-grown and wild foods were associated with safety, health, strength and vitality, but care must be taken to separate home-grown crops from the commercial crops sprayed with agrochemicals. One interviewee now avoids ordering certain dishes in informal market restaurants after experiencing throat irritation following a meal there, which she linked to agrochemicals. Another interviewee shared an experience of permanently losing hearing in one ear after eating market pork, which the doctor linked to a disease affecting pigs at the time. A local government health official confirmed that *Staphylococcus suis* in infected pork can cause hearing loss and is common in the study area. Other food safety concerns involved hygiene and lack of freshness.

#### 3.3.2. Healthiness

Sourcing foods from wild–cultivated food environments was associated with greater strength and vitality, whereas market foods were associated with pain, weakness and fatigue: “*When you eat home-grown food*, *you are stronger. You have no aches and pains. Your joints*, *your wrists*, *do not have any pain at all. If you eat food from down below [in town]*, *you will start to have symptoms like headaches and fatigue*” (Interviewee in her early 60s). One interviewee attributed aches and pains to consuming market meat, rather than self-raised animal meat: “*I do not know exactly why*, *but when I eat food from town*, *I get aches and pains. Like when my child buys chicken in town*, *I get aches in my body. If I eat my own chickens*, *I don’t get headaches*” (Interviewee in her early 60s). Interviewees concurred with the focus group participants that natural food environments were healthier food sources than market food environments.

#### 3.3.3. Affect

Natural food environments were generally associated with positive affective experiences. Interviewees expressed joy over foraging in wild forest environments: “*I love foraging for mushrooms*, *catching fish and foraging for wild foods*” (Interviewee in her late 30s). One interviewee recounted a positive experience taking the children to play in the streams while catching fish, followed by a picnic in the forest: “*Catching fish is like going on a trip to play in the water… It was fun. We packed our lunch and chatted while eating*” (Interviewee in her late 30s). Social foraging with friends can also offset feelings of exhaustion from walking in the forest for hours: “*When I go to collect banana blossoms and Meliantha suavis*, *I have to walk… But if I go with friends*, *even though I get tired*, *I still feel good*” (Interviewee in her early 20s). Foraging as a group can also allay fears over ghosts when walking through sections of the forest believed to be haunted: “*Going with friends is more fun*, *because we get to talk. Most of the time*, *I’m afraid of ghosts… I’m afraid*, *because someone committed suicide there*, *and people buried bodies in that area. So*, *I don’t dare to go alone*” (Interviewee in her early 20s). Wild–cultivated swiddens were also associated with positive feelings of peaceful calm and finding a refuge from the noise in the village: “*If I stay at home*, *I hear all kinds of noises and am not content. If I go over there [to the rotational farmland]*, *I am content*” (Interviewee in her early 60s). The interview data corroborated the focus group decision matrix results, where natural and social environments were ranked as “good” or “very good” for affect. Market environments were associated with mixed affective responses. One interviewee reported feeling good at seeing an abundance of food, followed by negative emotions over not being able to afford the available food items (Interviewee in her late 30s). Informal markets were ranked as “average” in the decision matrix, reflecting the mixed positive and negative affect experienced at the fresh market.

#### 3.3.4. Convenience

Convenience refers to individuals’ time and energy expenditures. Interviewees reported travel times and modes of transportation to different types of food environments as follows: (i) wild–cultivated home gardens around the home arriving instantly by foot, (ii) wild–cultivated agricultural streams—by foot or at most 20 min by motorbike, (iii) wild–cultivated rotational farmlands—by foot or maximum half an hour travel time by motorbike, (iv) foraging in wild forest food environments—2 h by foot, (v) village kiosk—1–5 min walk, and (vi) market trip to town by car or motorbike—between 2 and 3 h to an entire day. Convenience involves more than travel time; preparation and storage are also involved. One interviewee noted that foraged wild foods can be stored for several meals to prepare simple dishes like fish and chili paste, making them convenient: “*Natural foods are quite convenient*, *because I live in the mountains*, *right? So*, *if we’re free for a day*, *we’ll go and forage to get some wild foods. For example*, *if I go fishing*, *I won’t just catch some fish. I’ll also get some vegetables too. In the rainy season*, *I will get fiddlehead ferns*, *bamboo shoots*, *or pumpkin stems. This way*, *we have enough food for several meals… We store what we gather*, *and when we go to work in the morning*, *we’ll make a simple meal*, *such as boiled fish and chili paste*” (Interviewee in her late 30s). Others noted the inconvenient amount of time required to find desirable forest foods: “*Sometimes*, *for certain vegetables*, *we want to eat them*, *but they’re far away*, *so we have to take time to walk and find them*” (Interviewee in her early 40s). Preparation of some forest foods, such as wild banana flowers, can also take longer but is worthwhile due to the superior flavor: “*Wild banana blossom tastes better [than domesticated banana], but it is more fibrous. Before cooking*, *after cutting it*, *we need to take away this string-like residue. If we don’t take it out*, *it can clog our throat. Domesticated banana blossom does not have these fibers*” (Interviewee in her 50s).

Another interviewee expressed the convenience of a home garden, as compared to markets: *“Staying at home is convenient… Things like galangal, lemongrass, kaffir lime leaves*—*we grow them all at home. It’s much more convenient than going to the market. A trip to the market takes up the whole day”* (Interviewee in her early 40s). This interviewee also expressed the cost-effectiveness of growing perennials in wild–cultivated food environments with long-term returns from initial energy–time investments: *“We grow it ourselves, so it takes time and effort, but it lasts, and we can eat it for many years. We only have to put in the effort for the first year after planting. We take care of it, and then we can enjoy it for several years without wasting effort. It’s cost-effective”* (Interviewee in her early 40s). The energy and time inputs in wild–cultivated food environments were evaluated favorably by interviewees, helping explain the “very good” ranking for convenience of wild–cultivated food environments in the decision matrix.

Markets were considered convenient in that they require little energy expenditure to acquire the foods, as compared to walking in the forest; however, this convenience was outweighed by their unaffordability: “*[The market] is convenient*, *because if you have money*, *you can just buy whatever you want there—a lot easier than going into the jungle and looking for food. But since I don’t have any money*, *it is necessary for me to find food in the forest*” (Interviewee in her 30s). Other interviewees considered markets inconvenient due to travel times: “*As for food… if I go to buy it from the market*, *I have to go all the way there*, *and it’s quite far. If you ask me*, *it would be convenient if the market were closer to our neighborhood*, *but it’s a bit too far from here*, *so it’s not very convenient*” (Interviewee in her late 30s). The mixed reviews of market convenience with low energy expenditures but high travel times help explain why informal markets were ranked as “average” for convenience (2.8 out of 5) in the decision matrix by focus group participants.

#### 3.3.5. Accessibility

Considering distance as a simple proxy for access, one interviewee compared the distances to different types of food environments as follows: “*The market is far. The home garden and the forest are closer. The market is far. I have to ride my motorcycle there. For the forest*, *I just walk there*” (Interviewee in her 30s). The interviewee continued, “*Walking in nature is much easier*” (Interviewee in her 30s). Geolocated participant observation data confirmed the interview findings that formal and fresh markets were located farthest away (~24 km), followed by wild food environments (~870 m). Wild–cultivated food environments, such as home gardens and agricultural streams, were the closest on average (~159 m) (see Table 7).

**Figure 5:**
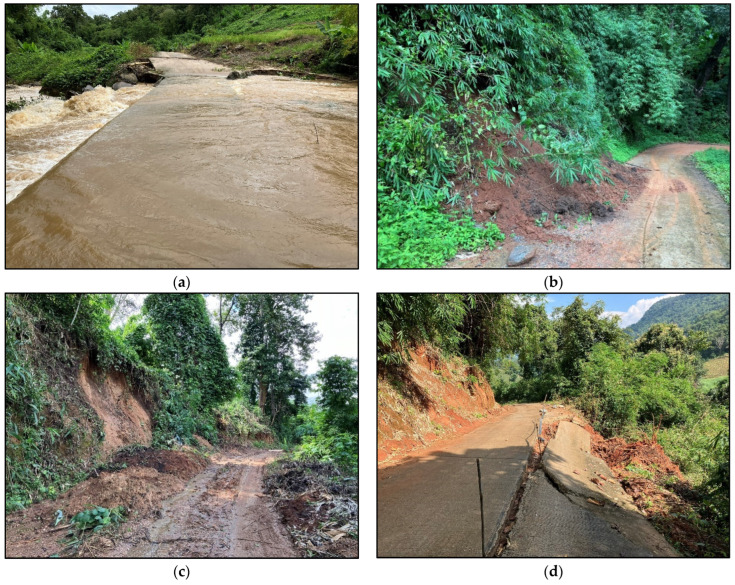
Difficulties accessing built market environments in rainy season: (**a**) seasonal periodic road flooding; (**b**) a landslide covering half the road during rainy season; (**c**) muddy road conditions on the paved road following landslides and heavy rains; and (**d**) a collapsed road following a landslide.

Distance is an imperfect measure of accessibility, because terrain and weather conditions also affect access. Participant observations confirmed the interviewees’ assertion that access varies by season and involves road and trail conditions that are heavily influenced by weather. For instance, the road from San Din Daeng to Chom Thong town (where the fresh market is located) can flood for several days (see Figure 5a), deteriorate with landslides and mud (see Figure 5b,c) and even collapse from excess rain and landslides (see Figure 5d) during the rainy season.

**Figure 6:**
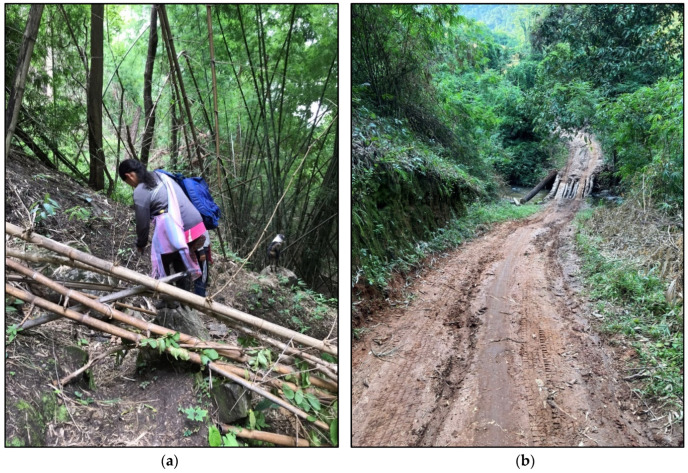
Access in natural food environments in rainy season: (**a**) difficult access with fallen bamboo, trees and vegetation in wild food environments; and (**b**) unpaved roads to rotational crop fields can become muddy and inaccessible for some vehicles. Slippery mud can pose a risk of falls and injuries in rainy season.

Rain can also transform unpaved roads and forest trails into slippery mud that can impede access to wild and some wild–cultivated food environments (see Figure 6b): “*If it rains*, *[the road to the rotational farm gets] a bit difficult*, *because the road gets slippery. It’s just red soil*, *so it gets slippery when wet*” (Interviewee in her early 20s). Poor access in rainy season encourages accessing wild–cultivated home gardens nearby: “*But now*, *with the rains and floods*, *we don’t go far anymore. We gather things around the back and sides of the house*, *growing them nearby*” (Interviewee in her early 40s). Fallen trees and bamboo thickets can also make access more difficult, although not impassable in wild food environments (see Figure 6a). Wild food environments are more accessible in the dry season when forest fires clear the forest floor.

Accessibility varies by age and health at the individual level. One interviewee in her 30s reflected on how poor health reduced the accessibility of wild food environments while she recovered from surgery: “*Now I can’t really do much. I can’t really go [to the forest] anymore. If I feel healthy*, *I can go anywhere*” (Interviewee in her 30s). Elderly respondents report high desirability of wild foods but inability to access wild and wild–cultivated environments due to reduced mobility at an advanced age. Not only community-level but also individual-level variation in the physical accessibility of food environments across different distances and terrains can be decisive in selecting one food environment over another. Kin and community are important for mediating access to natural food environments at an advanced age. All elderly interviewees described their children foraging and sharing “mountain foods” and mushrooms with them, now that they can no longer directly access wild food environments due to age-related mobility issues. Kin and community becomes an important social food environment for elderly community members to acquire foods via food sharing from otherwise inaccessible natural food environments.

#### 3.3.6. Availability, Affordability and Price

Seasonality was emphasized for both availability and access. The availability of wild and cultivated foods varied greatly between the dry, rainy and cold season. Availability in natural food environments varies not only by season but also by time of day and weather conditions. For instance, the availability of some nocturnal frogs and fish in wild and wild–cultivated food environments is limited to a specific season (transitional period between rainy and cold season), as well as the time of day (after 7:30 p.m. for some species; around 2 a.m. for other species) and weather (warm and no rain). Participants in both focus groups and interviews agreed that wild food environments are most abundant and plentiful in the rainy season. The peak harvesting time in wild–cultivated agricultural systems is in the cold season. Market reliance is highest during the dry season when wild, wild–cultivated and cultivated food environments are too dry to support substantial vegetative growth. Lower production and supply also increases the price of market vegetables in the dry season: “*The hardest time is dry season. Even at the market*, *the vegetables and food become more expensive*” (Interviewee in her early 20s).

The interviewees and focus group participants all considered natural food environments the most affordable and markets as the least affordable. Wild food environments were ranked as the most affordable and had “very good” affordability in the decision matrix—a ranking supported by interview data: “*All the food in the forest is free—you can collect everything*” (Interviewee in her early 20s). Wild–cultivated natural food environments were also ranked as having “very good” affordability by the focus group participants in the decision matrix, which is supported by interviewees’ perceptions: “*The natural foods that we grow ourselves are the cheapest*” (Interviewee in her early 40s) and “*One has no money*, *but one can eat from what one has planted and grown*” (Interviewee in her early 60s). Another interviewee specified the affordability of foods in her home garden: “*We grow galangal and lemongrass in our home garden. We don’t have to buy them*, *and they don’t cost any money. We grow them ourselves and eat them. That’s the cheapest*” (Interviewee in her early 40s).

Both formal and informal markets, meanwhile, were considered as low affordability, supporting the focus group rankings of “not good” for informal markets and “not good at all” for formal markets: “*The fresh market is the most expensive*” (Interviewee in her early 20s), and “*The most expensive places are shopping malls*, *like Tesco*, *7-Eleven*, *Tesco Lotus*, *Big C*, *and places like that*” (Interviewee in her early 40s).

Affordability, as a function of price in relation to income, is a key factor in food environment decision-making. Especially in low-income settings, the affordability of food items can shape consumer–food environment interactions. In San Din Daeng, the incomes of elderly residents or parents of young children relying solely on government social pensions or child support grants are as low as USD 17 (THB 600) per month. One three-person family received a combined annual income of USD 800 (THB 27,400) or USD 267 per person. With annual household incomes as low as USD 800, the price ceilings for affordable food items are low. Based on our market price comparison, the prices of food items were lower in informal fresh markets as compared to formal supermarkets across all the food groups (see Table 8; see the SI for a full table of the disaggregated food items), which can explain why informal markets were ranked slightly better than formal markets in the decision matrix.

#### 3.3.7. Cultural Appropriateness

Pgaz K’Nyau cultural foods were strongly tied to wild natural food environments. Pgaz K’Nyau wild foods were described as highly desirable but also difficult to find, requiring foraging skills and knowledge: “*There are many Pgaz K’Nyau foods. There are vegetables…I like everything. All the wild food. But it’s hard to come by. If I can find it*, *I like to eat it all*” (Interviewee in her 30s). Wild foods, such as wild banana flowers (*Musa spp.*), were listed as ingredients for cultural dishes like Pgaz K’Nyau rice porridge (*tha phoh phoh*). Highland subsistence rice from wild–cultivated swiddens is closely bound to Pgaz K’Nyau cultural identity, unlike jasmine rice from lowland markets. The interview findings corroborate the “good” and “very good” focus group rankings for the cultural appropriateness of wild and wild–cultivated natural food environments as compared to the “not good” and “not good at all” evaluations for informal and formal markets.

### 3.4. Decision-Making Trade-Offs and Value Negotiations

Unequal weighting of different characteristics impacts how decisions are made when confronted with decision-making trade-offs. Flavor was the evaluation criterion ranked most highly by focus group participants (see Table 3). While flavor was weighted 4.6 out of 5, convenience was ranked lower at 4.1 out of 5, explaining why community members may choose to expend time and energy (an inconvenience) foraging for highly flavorful forest foods. For instance, in the late dry season, the highly desirable barometer earthstar mushroom (*Astraeus hygrometricus*) emerges from the forest floor. The flavor of this firm-textured mushroom outweighs the inconvenience (time and energy investment) of hiking through the forest, resulting in an individual’s decision to interact with a wild forest environment rather than purchasing earthstar mushrooms at the fresh market at a high price. Moreover, focus group participants and interviewees describe the enjoyability of foraging in the forest with friends and family. The inconvenience of strenuous hiking with difficult access up steep terrain is offset by the positive affect of enjoyable socializing. Similarly, decision-making trade-offs where flavor, food safety, affect and affordability outweigh convenience help explain one interviewee’s decision to wake up at two in the morning to wade in streams catching nocturnal frogs and fish, rather than wake up at a convenient hour to go food shopping at the market.

Decision-making trade-offs in wild–cultivated food environments include labor and time investments (inconvenience). Growing rice in rotational croplands requires a large amount of time and labor. However, both time and labor are free of financial cost due to reciprocal labor practices in San Din Daeng village. Purchasing sufficient rice for the year would cost ~THB 10,000 for the average family, which few families can afford. The desirability (taste, healthiness, safety, freshness) and affordability of growing rice in wild–cultivated food environments outweighs the inconvenience caused by time and labor investments. As one interviewee explained:

*“The rice we grow ourselves is sweeter and more fragrant. When we buy rice from the market, it’s bland, and I feel like the nutrients are gone. Jasmine rice is expensive, but it doesn’t taste as good, and the vitamins are lost in the process of preservation. We don’t know how long the rice in the market has been stored. I’ve already experienced that, so I prefer to grow our own rice. It’s worth the effort. We only have to work hard for one year, and we get to eat for two to three years. Next year, I might plant less, because this year we grew a lot and had a larger harvest. I’ll plan and calculate how much rice is left… If we get a good harvest this year, we can plant less next year and make it easier for ourselves. It depends on the family”* (Interviewee in her early 40s).

Even labor-intensive rice production in wild–cultivated food environments was considered worthwhile in the decision-making calculus for the desirability (taste, food safety, healthiness) and affordability of highland rice compared to market-purchased lowland jasmine rice. Almost all the households in San Din Daeng village are entirely self-sufficient with highland rice, reflecting the labor–time–cost–flavor–health decision-making trade-offs in wild–cultivated food environments. Decision-making trade-offs and synergies are reflected in the decision matrix weights for different criteria and coincide with participant observation findings of individuals evaluating whether to shop, farm or forage.

## 4. Discussion

Understanding consumer decision-making and food choice in food environments is a critical first step toward a positive food system transformation to promote social and environmental well-being [72]. For the residents of San Din Daeng, natural food environments provided a safe, healthy, affordable, desirable and accessible food environment. Markets were mostly perceived as difficult to access, inconvenient, expensive and unsafe. The decision matrix and mixed methods approach outlined in this paper provided a simple and cost-effective way to better understand food acquisition decision-making in different types of food environments. The mixed methods approach helped contextualize the reasons behind the decision matrix rankings. The preference ranking focus group protocol and multi-criteria decision matrix output provide a rapid (<1.5 h) data collection instrument to assess food environment decision-making preferences in diverse contexts, including those undergoing food environment transitions from natural to built food environments.

### 4.1. Comparing Preference Ranking Decision Matrix Results to Reported Food Environment Use

The proposed multi-criteria decision matrix and mixed methods approach assesses individuals’ subjective evaluations of food environment types and characteristics. Subjective perceptions and preferences may not reflect actual behavior. Perception–behavior gaps (sometimes referred to as intention–behavior or attitude–behavior gaps) have been observed in a range of decision-making contexts from organic food purchasing [73], fast-food decision-making [74], food waste [75], to ethical consumerism [76]. Structural factors constraining individual agency in food acquisition through poverty [77], racism [78,79,80,81,82], neoliberalism [83] and settler colonialism [84,85,86] in low-income, rural or Indigenous contexts can exacerbate perception–behavior gaps. Individuals’ food choice options are also constrained by logistical operations, affordability, accessibility and socio-cultural beliefs that can contribute to perception–behavior gaps [87].

When possible, it is ideal to compare actual food environment use to decision matrix evaluations. In this study, we are able to compare the decision matrix and mixed methods results to the results from a diet quality and food environment use survey published earlier [67]. This comparison shows large congruencies between the reported food environment perceptions and actual food environment use at the study site. Wild–cultivated food environments were ranked most highly in the decision matrix, supporting our earlier survey results showing that more foods were acquired from wild–cultivated food environments than any other type of food environment (37% or 88 out of 240 food items consumed the previous day by 31 women) [67]. In the survey, women also reported the highest frequency of interactions with wild–cultivated environments (daily visits), as compared to wild (once a month), informal market (four times per week) and formal market (once a year) environments [67]. Formal market types of food environments were ranked most poorly across all the criteria in the decision matrix, coinciding with our previous survey results showing that none of the food items consumed the previous day in San Din Daeng were acquired from formal markets [67]. “Kin and community” were ranked as a “good” food environment and ranked third best out of five types of food environments, which aligns with the diet survey data, in which “kin and community” was the third most common source, with 15% of foods consumed the previous day acquired via food sharing (37 out of 240 food items) [67].

While the decision matrix aligned with previous diet survey results in the majority of cases, the decision matrix did not reflect the frequency of consumption of foods from informal markets and wild food environments. Wild food environments were ranked second highest and “good” overall, yet only 8% of food items (18 out of 240 food items) consumed the previous day were acquired from wild food environments [67]. Informal markets were ranked second worst and as “not good” overall, yet 36% of foods consumed the previous day (87 out of 240 food items) were acquired from informal markets [67]. This could be because subjective weighting might underestimate the time constraints for foraging or the importance of readily available and convenient dry and canned market goods that are well suited for highland contexts without electricity and refrigeration. The village kiosk has made informal markets much more accessible and convenient, and participants ranked the village kiosk more highly than the fresh market in town (see the food environment preference ranking results in the SI). The informal market presence in the village could help explain why respondents report highly frequent interactions with informal markets (four times per week) and high informal market use despite the overall low ranking in the decision matrix results.

Other reasons why individuals might choose to continue shopping at informal markets that they perceive as “not good” or consume less forest foods despite perceiving wild food environments favorably could involve structural factors. Structural factors include national education policies that force Indigenous youth to attend lowland boarding schools, altering the time allocations for foraging and influencing youth food preferences that affect family eating and shopping habits [84]. Structural factors that might shape wild food environment use include national park regulations. Previous work has shown how restricted legal access to wild food environments can negatively affect wild food use [88]. Wild foods hold high cultural salience and value, especially for Indigenous Peoples [89,90,91], contextualizing why wild food environments might still be ranked favorably despite limited use.

### 4.2. Subconscious Factors in Food Choice

The discrepancy between the negative ranking for informal markets and the high reported market use also points to the subconscious nature of decision-making processes involved in food choice [6]. Individuals are not purely rational actors and tend to make decisions based on incomplete information, subjective impressions and evolutionary psychology [62]. Humans are evolutionarily predisposed to be biased toward high-energy and high-fat food sources [44,92]. As ultra-processed foods proliferate in modern food environments, individuals are more likely to navigate toward energy-dense foods in their built food environments [93,94]. Marketing strategies for ultra-processed foods can further trigger neurological pathways and cognitive biases toward readily available energy-dense foods in informal markets [95]. Snack companies’ marketing strategies are known for being particularly creative and innovative in Thailand [96] and are bolstered by structural barriers to food marketing reform [97]. E-commerce via internet and mobile data coverage can increase food advertisings’ outreach to rural populations [98]; however, the dietary impacts of food marketing research, and of neuromarketing in particular, in rural, LMIC contexts remain underdeveloped. Evolutionary implicit bias in humans’ spatial cognition around food, combined with subconscious marketing strategies, could help explain why individuals continue to shop at markets that go against their conscious value perceptions of markets as “not good”.

Taste is an important individual factor in food decision-making [99] and was weighted most highly by focus group participants. Sweet flavors and fatty textures often signify a high-energy food source and can trigger evolutionary opiodergic, dopaminergic and serotonergic neurological pathways that can improve mood and mitigate stress but also increase the risk of overweight, obesity and diabetes [44]. Intergenerational changing taste preferences from bitter flavors (associated with natural food environments) among elders to fatty and salty flavors (associated with markets) among younger generations are both an indicator and a mechanism of gradual food environment transitions from natural to built food environments (Pattern 3 in the Downs et al. [39] food environment transition framework). Our proposed food environment and taste preference ranking focus group activities can help tease out subtle processes of changing taste and food environment preferences in food environment transitions.

### 4.3. Limitations

The decision matrix and mixed methods approach is designed to collect perceptions and preferences of different types and characteristics of food environments. Conclusions about actual food environment use and behaviors can, therefore, not be drawn from the decision matrix outputs alone. Diet quality questionnaires with additional food environment use questions or direct observation of food environment use are needed to determine actual food environment use and individuals’ behaviors. The proposed decision matrix and mixed methods approach is best suited for studies focusing on preferences and perceptions or as a complementary method that can be integrated into larger studies that are interested in compiling food environment evaluations.

A limitation of the weighted sum decision matrix approach is that the same measurement units must be used (e.g., individuals’ preferences on a Likert scale from 1 to 5) [5]. The weighted sum model, thus, cannot be integrated with objective measures of characteristics in Turner et al.’s [40] external domain (e.g., price in dollars, vendor and product properties by bacterial concentrations in fresh produce). Another limitation is that subjective weighting relies on individuals’ perceptions, which are challenging to standardize between individuals (for instance, some individuals might rank “very good” much more strictly than others). Subjective weighting is sometimes referred to as the “Achilles heel” of multi-criteria decision analysis; however, eliminating weights is equally arbitrary and subjective [61]. Given that the proposed method focuses on individuals’ preferences and perceptions, we consider the subjective weights useful data that can help explain individuals’ priorities as they navigate diverse food environments. A different approach would be to use an automatic democratic method for inductively generating weights from within the data. Future studies could further develop and improve weighting techniques for food environment decision-making matrices.

### 4.4. Strengths of Mixed Methods

The mixed methods strengthened the results through triangulation and providing more in-depth information [100,101,102]. Triangulation is recommended when studying the complex and oftentimes subconscious decision-making processes behind food choice in food environments [41]. Triangulation of the focus group decision matrix results with the mixed methods found generally consistent results from different data types. The qualitative interview data proved valuable for interpreting the food environment preference ranking scores and understanding why wild–cultivated food environments were the most preferred and markets were the most disliked. Interviewees emphasized the multi-attribute decision-making synergies between food safety, flavor, affect, healthiness, convenience, affordability and accessibility in natural food environments. Food safety was repeatedly mentioned as a concern in market food environments, with community members expressing distrust over agrochemicals and disease. Poor quality texture and flavor were leveraged as additional food safety indicators. Beliefs, attitudes and meanings surrounding food safety are known to shape consumer food choice in different contexts, including in LMICs [103]. Farmers in India, for instance, have expressed similar concerns over “inflammatory agricultures” and perceive agrochemicals as reducing the nutritional value, flavor and healthiness of food crops [104]. Advances in embodied political ecologies of health are centering the body in discussions of body–health interlinkages in relation to both nutrition and contamination that could inform future food environment research focusing on food quality and desirability [105,106,107,108,109].

Multi-criteria decision analysis carries widespread applications in public health. Previous multi-criteria decision analysis techniques have been used to design health and nutrition interventions [110,111], stunting prevention [112], diabetes management strategies and other meal planning [113,114], and sustainable diet design [115]. Our decision matrix and mixed methods approach complements previous multi-criteria assessment approaches for diet quality and public health. The multi-criteria decision matrix method can be embedded within broader food environment and diet assessments to quantify individuals’ evaluations of food environments. The approach offers a simple and fast method for food environment researchers and public health practitioners interested in understanding how individuals or patients view their food environments—perceptions that influence food choice and diet quality, with broader implications for public health.

## 5. Conclusions

Daily decision-making shapes the places visited and the types of foods obtained, which in turn shapes consumption. Understanding food decision-makers’ preferences for the characteristics of different types of food environments provides key insights into the decision-making processes behind people’s interactions with different food environments. Whilst impossible to capture the complexity of daily micro-decision-making in diverse food environments entirely, this paper presents a simplified decision matrix and mixed methods approach for evaluating multiple decision-making characteristics across different types of food environments. By combining different food environment frameworks into a single multi-criteria decision matrix using a weighted sum model [39,40,45,65], we synthesize multiple conceptual frameworks into a single data collection instrument that can be used to assess perceptions of decision-making factors for different types of food environments in diverse contexts. We used qualitative and quantitative mixed methods to triangulate the decision matrix results and found generally consistent results between different data types, with large congruencies between the decision matrix, mixed methods and previously published survey data. Our results also highlight the importance and value of natural food environments for Indigenous communities. Considerations of flavor and food safety can be prioritized by food environment researchers and practitioners working with Indigenous communities concerned about contamination. Policymakers and public health experts could promote Indigenous and rural populations’ access to natural food environments as a preferred healthy and affordable source of quality foods. The mixed methods, multi-criteria decision matrix and preference ranking protocol offers a low-cost and rapid food environment assessment approach for studying dynamic Indigenous and rural food environment transitions in diverse LMIC contexts. The proposed methodology is fast, flexible and fun, and can accommodate the ever-evolving theoretical developments in food environment research.

## Figures and Tables

**Table 1:** Mixed methods for assessing decision-making in food environments.

Characteristic	Method	Measure	Output
Access	(1) Focus group—preference ranking	Likert scale	Decision matrix
	(2) Geolocated participant observation	Distance	Community food environment map
	(3) Focus group—participatory mapping (4) Qualitative interviews	Distance Quotes	Community food environment map Key quotations
Affordability/Price	(1) Focus group—preference ranking	Likert scale	Decision matrix
	(2) Market price comparison	Price	Price comparison
	(3) Qualitative interviews	Quotes	Key quotations
Convenience	(1) Focus group—preference ranking	Likert scale	Decision matrix
	(2) Participant observation	Time	Time comparison
	(3) Qualitative interviews	Quotes	Key quotations
Availability and Desirability	(1) Focus group—preference ranking	Likert scale	Decision matrix
	(2) Qualitative interviews	Quotes	Key quotations

**Table 3:** Weighted sum multi-criteria decision matrix based on the focus group participants’ average ranked preferences in San Din Daeng village, using the expanded focus group preference ranking protocol (n = 16 in 2 focus groups; 44% household coverage).

Food Environment Characteristic	Weight *	Wild	Wild– Cultivated	Informal Market	Formal Market	Kin and Community
Affordability	3.8	4.9	4.8	2.3	1.4	4.7
Convenience	4.1	4.4	4.5	2.8	1.4	4.5
Accessibility	4.1	4.4	4.5	2.7	1.2	4.3
Availability	3.4	4.1	4.3	3.1	1.6	3.9
Desirability	4.0	4.6	4.7	2.8	1.5	4.0
Taste	4.6	4.8	4.7	2.9	1.8	4.7
Satiety	4.3	4.8	4.7	2.8	1.8	4.7
Affect	4.3	4.3	4.6	3.3	1.8	4.6
Safety	4.4	5.0	4.8	2.6	2.2	4.5
Freshness	3.8	5.0	4.9	2.5	1.8	4.3
Healthiness	4.2	4.9	4.8	2.7	2.1	4.5
Sustainability	3.8	4.9	4.7	2.8	1.9	4.1
Culture	3.8	4.4	4.6	2.2	1.3	4.1
Weighted Sum	4.0	3.7	3.8	2.2	1.4	3.5

* 1 = Not good at all (red). 2 = Not good. 3 = Average 4 = Good 5 = Very good (green). Cell colors represent preferences, with green most preferred and red least preferred. Note: The final weighted sum has been divided by the number of criteria to normalize the results on a scale from 1 to 5.

**Table 4:** Weighted sum model averages for different types of food environments, disaggregated by age group (n = 16 in 2 focus groups).

Participant Age Groups	Wild	Wild– Cultivated	Informal Market	Formal Market	Kin and Community
<35 years old	3.5	3.6	2.1	1.4	2.8
35–<45 years old	3.7	3.8	2.3	1.5	3.7
45–<55 years old	3.6	3.4	2.1	1.2	3.4
>55 years old	4.3	4.3	2.4	1.1	4.3

1 = Not good at all. 2 = Not good. 3 = Average. 4 = Good. 5 = Very good. Cell colors represent preferences, with green most preferred and red least preferred.

**Table 5:** Focus group participants’ taste preferences with the averages disaggregated by age category (n = 16 in 2 focus groups with 8 participants per focus group).

Participant Age Groups	Bland	Sour	Spicy	Bitter	Sweet	Fatty	Salty	MSG
<35 years old	3.0	3.0	2.8	3.0	2.8	4.0	3.8	4.3
35–<45 years old	4.0	3.2	3.2	3.3	3.0	3.2	2.5	3.8
45–<55 years old	4.0	3.5	2.5	4.5	2.0	2.0	3.0	3.0
>55 years old	3.0	2.8	3.8	4.8	2.0	2.0	1.8	1.5
Average	*3.6*	*3.7*	*3.4*	*3.6*	*2.4*	*3.0*	*2.7*	*3.3*

1 = Not good at all. 2 = Not good. 3 = Average. 4 = Good. 5 = Very good. Cell colors represent preferences, with green most preferred and red least preferred.

**Table 6:** Different food safety concerns mentioned by interviewees.

Food Safety Concern	Key Quotations
Agrochemical Residue Contamination	“Pesticides are used for almost everything. When we buy fruits, we wonder if they are pesticide-free”. (Interviewee in her early 40s)
	“If I go to Chom Thong [market in town] and see vegetables that are green, lush and beautiful, I don’t buy them”. (Interviewee in her 50s)
	“When we buy the vegetables, we’ll check if there’s any spots that the insects have bitten, whether they ate the leaf or not to ensure they’re pesticide-free. If they look very beautiful and fresh, then they sprayed pesticide on those… Some people, they don’t understand. They would be like ‘oh this vegetable looks very good’. But actually, the beautiful ones are likely dangerous. It’s best to choose the ugly ones with insect bites. They are safer” (Interviewee in her late 30s).
Inflammatory effects of agrochemicals	“One time, I ate fried rice with deep-fried pork at a restaurant in Chom Thong. They added Chinese kale. While I was eating, I felt the taste was strange and bitter, so bitter that it burned my throat. I came home with a sore throat. From then on, I never ate fried rice with deep-fried pork and Chinese kale again” (Interviewee in her 50s).
Disease	“I had an earache after eating pork in town. At that time, there was some kind of disease going around. I ate that pork, and it made me deaf. Now I can only hear from one side” (Interviewee in her early 60s).
Hygiene	“Yes, when I go to the city, I don’t eat from there, not unless I am really hungry. I usually eat at home or buy ingredients and take them back home to eat. I do not trust food from the restaurant—whether it is clean or not. It is, I guess, my personal preference” (Interviewee in her 50s).
Poor Storage	“We don’t know how long the rice in the market has been stored” (Interviewee in her early 40s).

**Table 7:** Average distance and travel time (one-way) to different types of food environments, based on geolocated participant observation data.

Food Environment Type	Average Distance (m)	Travel Time (min)
Wild	870	40
Wild–Cultivated	159	5–10
Informal Market in Village	192	5–10
Informal Market in Town	23,650	45

**Table 8:** Price comparison of formal and informal markets of built food environments in Chom Thong town, aggregated by food group.

Food Group	Formal Market (Price in USD/kg) ^1^	Informal Market (Price in USD/kg) ^1^	Difference in Price (%)
Fruits	1.82	1.07	52
Dark Green Leafy Vegetables	2.91	0.99	99
Vitamin A-Rich Fruit and Veg	1.57	0.81	63
Other Vegetables	2.17	1.31	49
Meat	3.90	3.09	23
Seafood	7.42	5.95	22
Eggs ^γ^	4.96	4.08	20

^1^ Price is in US dollars (USD), converted from Thai Baht (THB) at the exchange rate on the day of the price comparison. ^γ^ Price is per kilogram, except for eggs, which is the price for a pack of 30 eggs.

## Data Availability

The focus group preference ranking and market price comparison data are available in the Supplementary File S2: Food Environment Preference Ranking Results; File S3: Taste Preference Ranking Results, and File S4: Market Price Comparison Results. All the data will be made available in a data repository within one year of the end of the research project in accordance with National Science Foundation guidelines.

## Supplementary Materials

The following supporting information can be downloaded at https://www.mdpi.com/article/10.3390/ijerph22050711/s1, File S1: Focus Group Protocol and Data Collection Sheets; File S2: Food Environment Preference Ranking Results; File S3: Taste Preference Ranking Results; File S4: Market Price Comparison Results.

## Appendix A

**Table A1:** A sample weighted decision matrix to show how the weighted sum model is calculated using Downs et al.’s [39] and Herforth and Ahmed’s [65] food environment frameworks.

Criteria (C) Food Environment Characteristic	Weight (W)	Option A_1_: Wild Food Environment	Option A_2_: Cultivated Food Environment	Option A_3_: Informal Market	Option A_4_: Formal Market
Availability (C_1_)	W_1_	A_1_ × C_1_	A_2_ × C_1_	A_3_ × C_1_	A_4_ × C_1_
Affordability (C_2_)	W_2_	A_1_ × C_2_	A_2_ × C_2_	A_3_ × C_2_	A_4_ × C_2_
Accessibility (C_3_)	W_3_	A_1_ × C_3_	A_2_ × C_3_	A_3_ × C_3_	A_4_ × C_3_
Convenience (C_4_)	W_4_	A_1_ × C_4_	A_2_ × C_4_	A_3_ × C_4_	A_4_ × C_4_
Desirability (C_5_)	W_5_	A_1_ × C_5_	A_2_ × C_5_	A_3_ × C_5_	A_4_ × C_5_
Weighted Sum Model Score		= (A_1_ × C_1_ × W_1_) + (A_1_ × C_2_ × W_2_) + (A_1_ × C_3_ × W_3_) + (A_1_ × C_4_ × W_4_) + (A_1_ × C_5_ × W_5_)	= (A_2_ × C_1_ × W_1_) + (A_2_ × C_2_ × W_2_) + (A_2_ × C_3_ × W_3_) + (A_2_ × C_4_ × W_4_) + (A_2_ × C_5_ × W_5_)	= (A_3_ × C_1_ × W_1_) + (A_3_ × C_2_ × W_2_) + (A_3_ × C_3_ × W_3_) + (A_3_ × C_4_ × W_4_) + (A_3_ × C_5_ × W_5_)	= (A_4_ × C_1_ × W_1_) + (A_4_ × C_2_ × W_2_) + (A_4_ × C_3_ × W_3_) + (A_4_ × C_4_ × W_4_) + (A_4_ × C_5_ × W_5_)

**Table A2:** The pilot decision matrix (n = 8 in one focus group).

Food Environment Characteristic	Wild	Wild–Cultivated	Informal Market	Formal Market
Affordability	4.8	4.7	1.6	1.0
Convenience	3.8	4.1	3.1	1.1
Access	4.3	4.3	2.8	1.0
Availability	3.6	4.0	3.4	1.8
Desirability	4.4	4.7	2.8	1.3
Average	4.4	4.5	2.2	1.2

1 = Not good at all. 2 = Not good. 3 = Average 4 = Good 5 = Very good. Cell colors represent preferences, with green most preferred and red least preferred. Note: The final weighted sum has been divided by the number of criteria to normalize the results on a scale from 1 to 5.

## References

[1] Edwards W. (1954). The theory of decision making. Psychol. Bull..

[2] Einhorn H.J., Hogarth R.M. (1981). Behavioral Decision Theory: Processes of Judgement and Choice. Annu. Rev. Psychol..

[3] Kleindorfer P.R., Kunreuther P.J.H., Shoemaker H.C. (1993). Decision Sciences: An Integrative Perspective.

[4] Marler R.T., Arora J.S. (2010). The weighted sum method for multi-objective optimization: New insights. Struct. Multidiscip. Optim..

[5] Triantaphyllou E. (2000). Multi-Criteria Decision Making Methods. Multi-Criteria Decision Making Methods: A Comparative Study [Internet].

[6] Sobal J., Bisogni C.A. (2009). Constructing food choice decisions. Ann. Behav. Med..

[7] Marsh K., Goetghebeur M., Thokala P., Baltussen R. (2017). Multi-Criteria Decision Analysis to Support Healthcare Decisions. Multi-Criteria Decision Analysis to Support Healthcare Decisions.

[8] Tiwari D.N., Loof R., Paudyal G.N. (1999). Environmental-economic decision-making in lowland irrigated agriculture using multi-criteria analysis techniques. Agric. Syst..

[9] Kotikot S.M., Kar B., Omitaomu O.A. (2020). A Geospatial Framework Using Multicriteria Decision Analysis for Strategic Placement of Reserve Generators in Puerto Rico. IEEE Trans. Eng. Manag..

[10] Mayorga-Martínez A.A., Kucha C., Kwofie E., Ngadi M. (2023). Designing nutrition-sensitive agriculture (NSA) interventions with multi-criteria decision analysis (MCDA): A review. Crit. Rev. Food Sci. Nutr..

[11] Adem Esmail B., Geneletti D. (2018). Multi-criteria decision analysis for nature conservation: A review of 20 years of applications. Methods Ecol. Evol..

[12] Cegan J.C., Filion A.M., Keisler J.M., Linkov I. (2017). Trends and applications of multi-criteria decision analysis in environmental sciences: Literature review. Environ. Syst. Decis..

[13] Huang I.B., Keisler J., Linkov I. (2011). Multi-criteria decision analysis in environmental sciences: Ten years of applications and trends. Sci. Total Environ..

[14] Ferla G., Mura B., Falasco S., Caputo P., Matarazzo A. (2024). Multi-Criteria Decision Analysis (MCDA) for sustainability assessment in food sector. A systematic literature review on methods, indicators and tools. Sci. Total Environ..

[15] Gésan-Guiziou G., Alaphilippe A., Aubin J., Bockstaller C., Boutrou R., Buche P., Collet C., Girard A., Martinet V., Membré J.M. (2020). Diversity and potentiality of multi-criteria decision analysis methods for agri-food research. Agron. Sustain. Dev..

[16] Karlsson Potter H., Röös E. (2021). Multi-criteria evaluation of plant-based foods –use of environmental footprint and LCA data for consumer guidance. J. Clean. Prod..

[17] Ali B.M., Andersson M.G., van den Borne B.H.P., Focker M., van der Fels-Klerx H.J. (2022). Multi-Criteria Decision Analysis in Food Safety Risk Management: The Case of Dioxins in Baltic Fish. Foods.

[18] Ruzante J.M., Grieger K., Woodward K., Lambertini E., Kowalcyk B. (2017). The use of multi-criteria decision analysis in food safety risk-benefit assessment. Food Prot. Trends.

[19] Mazzocchi M., Ragona M., Zanoli A. (2013). A fuzzy multi-criteria approach for the ex-ante impact assessment of food safety policies. Food Policy.

[20] Fazil A., Rajic A., Sanchez J., McEwen S. (2008). Choices, choices: The application of multi-criteria decision analysis to a food safety decision-making problem. J. Food Prot..

[21] Abakarov A., Sushkov Y., Mascheroni R.H. (2013). A multi-criteria optimization and decision-making approach for improvement of food engineering processes. Int. J. Food Stud..

[22] Yeung Y.H., Lin R., Liu Y., Ren J. (2020). 3R for food waste management: Fuzzy multi-criteria decision-making for technology selection. Waste-to-Energy: Multi-Criteria Decision Analysis for Sustainability Assessment and Ranking.

[23] Romero-Perdomo F., González-Curbelo M.Á. (2023). Integrating Multi-Criteria Techniques in Life-Cycle Tools for the Circular Bioeconomy Transition of Agri-Food Waste Biomass: A Systematic Review. Sustainability.

[24] Martin D.S., Orive M., Martínez E., Iñarra B., Ramos S., González N., de Salas A.G., Vázquez L., Zufía J. (2017). Decision Making Supporting Tool Combining AHP Method with GIS for Implementing Food Waste Valorisation Strategies. Waste Biomass Valorization.

[25] Kesharwani N., Bajpai S. (2020). Batch anaerobic co-digestion of food waste and sludge: A multi criteria decision modelling (MCDM) approach. SN Appl. Sci..

[26] Angelo A.C.M., Saraiva A.B., Clímaco J.C.N., Infante C.E., Valle R. (2017). Life Cycle Assessment and Multi-criteria Decision Analysis: Selection of a strategy for domestic food waste management in Rio de Janeiro. J. Clean. Prod..

[27] Iacovidou E., Voulvoulis N. (2018). A multi-criteria sustainability assessment framework: Development and application in comparing two food waste management options using a UK region as a case study. Environ. Sci. Pollut. Res..

[28] Abu R., Aziz M.A.A., Noor Z.Z. (2021). Integrated Life Cycle Assessment, Life Cycle Costing and Multi Criteria Decision Making for Food Waste Composting Management. J. Adv. Res. Bus. Manag. Stud..

[29] Ren J., Toniolo S. (2020). Life cycle sustainability prioritization of alternative technologies for food waste to energy: A multi-actor multi-criteria decision-making approach. Waste-to-Energy: Multi-Criteria Decision Analysis for Sustainability Assessment and Ranking.

[30] Babalola M.A. (2015). A multi-criteria decision analysis of waste treatment options for food and biodegradable waste management in Japan. Environments.

[31] Magalhães V.S.M., Ferreira L.M.D.F., Silva C. (2022). Prioritising food loss and waste mitigation strategies in the fruit and vegetable supply chain: A multi-criteria approach. Sustain. Prod. Consum..

[32] Prišenk J., Turk J. (2022). Assessment of Concept between Rural Development Challenges and Local Food Systems: A Combination between Multi-Criteria Decision Analysis and Econometric Modelling Approach. Sustainability.

[33] Linnemann A.R., Hendrix E.M.T., Apaiah R., Van Boekel T.A.J.S. (2015). Food chain design using multi criteria decision making, an approach to complex design issues. NJAS—Wagening. J. Life Sci..

[34] Arslan E., Dedebaş T., Hastaoğlu E. (2024). Application of Multi-criteria Decision Making Techniques in Sensory Evaluation. Food Analogues: Emerging Methods and Challenges.

[35] Dogan M., Aslan D., Aktar T., Goksel Sarac M. (2016). A methodology to evaluate the sensory properties of instant hot chocolate beverage with different fat contents: Multi-criteria decision-making techniques approach. Eur. Food Res. Technol..

[36] Gurmeric V.E., Dogan M., Toker O.S., Senyigit E., Ersoz N.B. (2013). Application of Different Multi-criteria Decision Techniques to Determine Optimum Flavour of Prebiotic Pudding Based on Sensory Analyses. Food Bioproc. Technol..

[37] Mohammadian Mosammam H., Sarrafi M., Tavakoli Nia J., Mosammam A.M. (2017). Measuring Food Deserts via GIS-Based Multicriteria Decision Making: The Case of Tehran. Prof. Geographer..

[38] Vedovato G.M., Rehman Z.N., Bunzl N.B., Trude A.C.B. (2025). Food sources and acquisition by consumers of low income in urban neighborhoods: A conceptual framework and food decision tree. Appetite.

[39] Downs S.M., Ahmed S., Fanzo J., Herforth A. (2020). Food environment typology: Advancing an expanded definition, framework, and methodological approach for improved characterization of wild, cultivated, and built food environments toward sustainable diets. Foods.

[40] Turner C., Aggarwal A., Walls H., Herforth A., Drewnowski A., Coates J., Kalamatianou S., Kadiyala S. (2018). Concepts and critical perspectives for food environment research: A global framework with implications for action in low- and middle-income countries. Glob. Food Secur..

[41] Blake C.E., Frongillo E.A., Warren A.M., Constantinides S.V., Rampalli K.K., Bhandari S. (2021). Elaborating the science of food choice for rapidly changing food systems in low-and middle-income countries. Glob. Food Secur..

[42] Furst T., Connors M., Bisogni C.A., Sobal J., Falk L.W. (1996). Food choice: A conceptual model of the process. Appetite.

[43] Wansink B., Sobal J. (2007). Mindless eating: The 200 daily food decisions we overlook. Environ. Behav..

[44] Leigh Gibson E. (2006). Emotional influences on food choice: Sensory, physiological and psychological pathways. Physiol. Behav..

[45] Farrell P., Reeve E., Johnson E., Farmery A.K., Patay D., Thow A.M., Wu J., Bogard J.R. (2024). Measuring characteristics of wild and cultivated food environments: A scoping review. BMC Med..

[46] Kelly B., Flood V.M., Yeatman H. (2011). Measuring local food environments: An overview of available methods and measures. Health Place.

[47] Lytle L.A., Sokol R.L. (2017). Measures of the food environment: A systematic review of the field, 2007–2015. Health Place.

[48] Osei-Kwasi H.A., Laar A., Zotor F., Pradeilles R., Aryeetey R., Green M., Griffiths P., Akparibo R., Wanjohi M.N., Rousham E. (2021). The African urban food environment framework for creating healthy nutrition policy and interventions in urban Africa. PLoS ONE.

[49] Ahmed S., Kennedy G., Crum J., Vogliano C., McClung S., Anderson C. (2021). Suitability of data-collection methods, tools, and metrics for evaluating market food environments in low-and middle-income countries. Foods.

[50] Downs S., Warne T., McClung S., Vogliano C., Alexander N., Kennedy G., Ahmed S., Crum J. (2024). Piloting Market Food Environment Assessments in LMICs: A Feasibility Assessment and Lessons Learned. Food Nutr. Bull..

[51] Marshall Q., Hewavidana B.H. (2024). Adaptation of a Food Environment Typology for Urban Sri Lanka. [Internet]. https://hdl.handle.net/10568/159857.

[52] Toure D., Herforth A., Pelto G.H., Neufeld L.M., Mbuya M.N.N. (2021). An Emergent Framework of the Market Food Environment in Low- And Middle-Income Countries. Curr. Dev. Nutr..

[53] Wertheim-Heck S.C.O., Raneri J.E. (2019). A cross-disciplinary mixed-method approach to understand how food retail environment transformations influence food choice and intake among the urban poor: Experiences from Vietnam. Appetite.

[54] Downs S.M., Ahmed S., Warne T., Fanzo J., Loucks K. (2022). The global food environment transition based on the socio-demographic index. Glob. Food Secur..

[55] Kuhnlein H.V., Receveur O. (1996). Dietary Change and Traditional Food Systems of Indigenous Peoples. Annu. Rev Nutr..

[56] Damman S., Eide W.B., Kuhnlein H.V. (2008). Indigenous peoples’ nutrition transition in a right to food perspective. Food Policy.

[57] Kuhnlein H.V., Receveur O., Soueida R., Egeland G.M. (2004). Arctic indigenous peoples experience the nutrition transition with changing dietary patterns and obesity. J. Nutr..

[58] Reyes-García V., Powell B., Díaz-Reviriego I., Fernández-Llamazares Á., Gallois S., Gueze M. (2019). Dietary transitions among three contemporary hunter-gatherers across the tropics. Food Secur..

[59] Dyer J.S. (2005). MAUT-multiattribute utility theory. Multiple Criteria Decision Analysis: State of the Art Surveys.

[60] Wątróbski J., Jankowski J., Ziemba P., Karczmarczyk A., Zioło M. (2019). Generalised framework for multi-criteria method selection. Omega.

[61] Poulsen L.K. (2022). Literature Review: Multi Criteria Assessment of Food-Based Systems. https://norsus.no/wp-content/uploads/AR-04.22-Literature-review-MCA-of-food-based-systems.pdf.

[62] Kahneman D. (2003). A Perspective on Judgment and Choice: Mapping Bounded Rationality. Am. Psychologist..

[63] Kahneman D., Tversky A. (1979). Prospect theory: An analysis of decision under risk. Econometrica.

[64] Normann A. (2012). Consumer Food Choice—How, Why and When? The Importance of Attitudes, Preferences, Information, Alarm and Other Factors Influencing Food Choice Situations. https://www.diva-portal.org/smash/get/diva2:944140/FULLTEXT01.pdf.

[65] Herforth A., Ahmed S. (2015). The food environment, its effects on dietary consumption, and potential for measurement within agriculture-nutrition interventions. Food Secur..

[66] Bogard J.R., Andrew N.L., Farrell P., Herrero M., Sharp M.K., Tutuo J. (2021). A typology of food environments in the Pacific region and their relationship to diet quality in Solomon Islands. Foods.

[67] Zeitler L., Downs S., Powell B. (2024). Adapting Food Environment Frameworks to Recognize a Wild-Cultivated Continuum. Front. Nutr..

[68] Downs S., Manohar S., Staromiejska W., Keo C., Say S., Chhinh N., Fanzo J., Sok S. (2024). Centering context when characterizing food environments: The potential of participatory mapping to inform food environment research. Front. Nutr..

[69] Njuki J., Eissler S., Malapit H., Meinzen-Dick R., Bryan E., Quisumbing A. (2022). A review of evidence on gender equality, women’s empowerment, and food systems. Glob. Food Secur..

[70] Wolgast E.H. (1958). Do Husbands or Wives Make the Purchasing Decisions?. J. Mark..

[71] Krizan F., Puljic N.P., Bognar Z.B. The role of women as purchase decision makers in the family. Proceedings of the 2023 Conference on Economic and Social Development.

[72] Rampalli K.K., Blake C.E., Frongillo E.A., Montoya J. (2023). Why understanding food choice is crucial to transform food systems for human and planetary health. BMJ Glob. Health.

[73] Ismael D., Ploeger A. (2020). The potential influence of organic food consumption and intention-behavior gap on Consumers’ subjective wellbeing. Foods.

[74] Downs J.S. (2013). Does ‘healthy’ fast food exist? the gap between perceptions and behavior. J. Adolesc. Health.

[75] Seo J.Y., Yoon S. (2022). Food waste perceptions: Vice versus virtue foods. J. Consum. Mark..

[76] Untarini N. (2020). Studying the Attitudes-Behavior Gap in Ethical Consumerism: A review of Research. J. Adm. Bisnis.

[77] Sahn D.E. (1988). The effect of food price and income changes on the acquisition of food by low-income households. Econ. Dev. Cult. Change.

[78] Power M. (2023). Whiteness, racism and colourblindness in UK food aid. Hunger, Whiteness and Religion in Neoliberal Britain.

[79] Reese A.M. (2018). “We will not perish; we’re going to keep flourishing”: Race, Food Access, and Geographies of Self-Reliance. Antipode.

[80] Reese A.M. (2019). Black Food Geographies: Race, Self-Reliance, and Food Access in Washington, DC.

[81] Bowen S., Elliott S., Hardison-Moody A. (2021). The structural roots of food insecurity: How racism is a fundamental cause of food insecurity. Sociol. Compass.

[82] Greene M., Houghtaling B., Sadeghzadeh C., De Marco M., Bryant D., Morgan R., Holston D. (2023). Nutrition interventions addressing structural racism: A scoping review. Nutr. Res. Rev..

[83] Alkon A.H., Mares T.M. (2012). Food sovereignty in US food movements: Radical visions and neoliberal constraints. Agric. Hum. Values.

[84] Ferguson C.E., Marie Green K., Switzer Swanson S. (2022). Indigenous food sovereignty is constrained by “time imperialism”. Geoforum.

[85] Rotz S., Xavier A.L., Robin T. (2023). “It wasn’t built for us”: The possibility of Indigenous food sovereignty in settler colonial food bureaucracies. J. Agric. Food Syst. Community Dev..

[86] Whyte K.P. (2016). Indigenous Food Sovereignty, Renewal and U.S. Settler Colonialism. The Routledge Handbook of Food Ethics.

[87] Dover R.V.H., Lambert E.V. (2016). ‘Choice Set’ for health behavior in choice-constrained settings to frame research and inform policy: Examples of food consumption, obesity and food security. Int. J. Equity Health.

[88] Olesen R.S., Powell B., Kilawe C.J., Rasmussen L.V. (2024). Food environment change on wild food consumption in rural Tanzania. Food Secur..

[89] Ahmed S., Warne T., Stewart A., Byker Shanks C., Dupuis V. (2022). Role of Wild Food Environments for Cultural Identity, Food Security, and Dietary Quality in a Rural American State. Front. Sustain. Food Syst..

[90] Bharucha Z., Pretty J. (2010). The roles and values of wild foods in agricultural systems. Philos. Trans. R. Soc. B Biol. Sci..

[91] Garibaldi A., Turner N. (2004). Cultural Keystone Species: Implications for Ecological Conservation and Restoration [Internet]. http://www.ecologyandsociety.org/vol9/iss3/art1.

[92] Schoener T.W. (1971). Theory of Feeding Strategies. Annu. Rev. Ecol. Syst..

[93] de Vries R., Boesveldt S., de Vet E. (2021). Locating calories: Does the high-calorie bias in human spatial memory influence how we navigate the modern food environment?. Food Qual. Prefer..

[94] de Vries R., de Vet E., de Graaf K., Boesveldt S. (2020). Foraging minds in modern environments: High-calorie and savory-taste biases in human food spatial memory. Appetite.

[95] Stasi A., Songa G., Mauri M., Ciceri A., Diotallevi F., Nardone G., Russo V. (2018). Neuromarketing empirical approaches and food choice: A systematic review. Food Res. Int..

[96] Hawkes C. (2006). Uneven dietary development: Linking the policies and processes of globalization with the nutrition transition, obesity and diet-related chronic diseases. Glob. Health.

[97] Phulkerd S., Ngqangashe Y., Collin J., Thow A.-M., Schram A., Schneider C.H., Friel S. (2022). Moving from silos to synergies: Strengthening governance of food marketing policy in Thailand. Glob. Health.

[98] Varlese M., Misso R., Koliouska C., Andreopoulou Z. (2020). Food, internet and neuromarketing in the context of well-being sustainability. Int. J. Technol. Mark..

[99] Clark J.E. (1998). Taste and flavour: Their importance in food choice and acceptance. Proc. Nutr. Soc..

[100] Axinn W.G., Pearce L.D. (2006). Mixed Method Data Collection Strategies.

[101] Cresswell J.W., Plano Clark V.L. (2011). Designing and Conducting Mixed Methods Research.

[102] Small M.L. (2011). How to conduct a mixed methods study: Recent trends in a rapidly growing literature. Annu. Rev. Sociol..

[103] Isanovic S., Constantinides S.V., Frongillo E.A., Bhandari S., Samin S., Kenney E., Wertheim-Heck S., Nordhagen S., Holdsworth M., Dominguez-Salas P. (2023). How Perspectives on Food Safety of Vendors and Consumers Translate into Food-Choice Behaviors in 6 African and Asian Countries. Curr. Dev. Nutr..

[104] Nichols C.E. (2023). Inflammatory agriculture: Political ecologies of health and fertilizers in India. Environ. Plan. E Nat. Space.

[105] Nichols C. (2025). Centering the body in agricultural development: Bridging conceptualizations of bodies-as-mechanism and bodies-as-affective. Soc. Sci. Med..

[106] Nichols C.E., Del Casino V.J. (2021). Towards an integrated political ecology of health and bodies. Prog. Hum. Geogr..

[107] Nichols C., Kampman H., van den Bold M. (2022). Forging just dietary futures: Bringing mainstream and critical nutrition into conversation. Agric. Hum. Values.

[108] Guthman J., Mansfield B. (2013). The implications of environmental epigenetics: A new direction for geographic inquiry on health, space, and nature-society relations. Prog. Hum. Geogr..

[109] Hayes-Conroy A., Hayes-Conroy J. (2015). Political ecology of the body: A visceral approach. The International Handbook of Political Ecology.

[110] Baltussen R., Niessen L. (2006). Priority setting of health interventions: The need for multi-criteria decision analysis. Cost Eff. Resour. Alloc..

[111] Aliasgharzadeh S., Ebrahimi-Mameghani M., Mahdavi R., Karimzadeh H., Nikniaz L., Tabrizi J.S., Pourali F. (2022). Prioritizing population-based nutrition-related interventions to prevent and control hypertension in Iran: A multi-criteria decision-making approach. BMC Med. Res. Methodol..

[112] Ainul Yaqin A.M., Rosyid M.J., Leksono V.A., Wantira A.D. (2024). A Preference-Oriented Multi-Criteria Decision Model for Stunting-Prevention Food Basket Ranking using AHP-TOPSIS. J. Ind. Eng. Res. Appl..

[113] Zadeh M.S.A.T., Li J., Alian S. Personalized Meal Planning for Diabetic Patients Using a Multi-Criteria Decision- Making Approach. Proceedings of the 2019 IEEE International Conference on E-Health Networking, Application and Services, HealthCom.

[114] Haseena S., Saroja S., Revathi T. (2022). A fuzzy approach for multi criteria decision making in diet plan ranking system using cuckoo optimization. Neural Comput. Appl..

[115] Bashiri B., Kaleda A., Vilu R. (2025). Integrating Multi-Criteria Decision-Making with Multi-Objective Optimization for Sustainable Diet Design. J. Clean. Prod..

