# Supplementary material for: Foraging, Farming or Shopping? A Decision Matrix Approach for Food Environment Assessments
Source: Int J Environ Res Public Health. 2025 Apr 30;22(5):711. doi: 10.3390/ijerph22050711 (PMC12110889; doi:10.3390/ijerph22050711)
Supplement: Supplementary file 1 [file ijerph-22-00711-s001.zip › S1 Focus Group Protocol and Data Collection Sheets.pdf]

## Focus Group Protocol

*Overview description*

This focus group protocol includes three activities: a food environment preference ranking activity, a taste ranking activity and a participatory mapping activity.

The purpose of the preference ranking activity is to assess subjective preferences of different types of food environments based on each food environment characteristic. We include a simplified (5 characteristics) and an expanded version (10 characteristics and 3 individual factors).

The taste preference ranking exercise is designed to capture individuals' preferences for different tastes and flavors, including: sweet, sour, spicy, salty, bland and bitter flavors. We include fatty as a texture linked to taste. For our focus group in Thailand, we included monosodium glutamate (MSG) as a flavor, because local participants distinguish between the flavor of MSG and salt. The taste preference ranking protocol can be locally adapted as needed. The purpose of the taste preference ranking activity is to collect data on intergenerational changing taste preferences in food environment transitions.

The participatory mapping activity is designed to help geolocate local food environments that can be entered into a Geographic Information System to estimate distances (as a proxy for access) to different food environments.

**Estimated time duration:** ~ 2 hours

### Preparation Prior to Focus Group

1. Conduct a focus group following Down et al.'s (2024) participatory food environment mapping protocol to identify local food environments.
2. Modify the data collection sheet at the end of this document to include the local food environments identified in that focus group and translate the names of local food environments into the local language.
3. Take photos of the different local food environments and replace the photos in the data collection sheets.
4. Print the locally adapted data collection sheets – at least one per participant.
5. Print A4-size screenshots from Google Maps and Google Earth (both satellite and street layouts) of the areas identified as local food environments in the previous food environment mapping focus group following the Downs et al. (2024) protocol.

### Materials Needed:

- Print-outs of data collection sheets (minimum 1 set per participant)
- Beans for participants who cannot write
- Pens for participants who can write + marker pens + white-out

**Facilitator Instructions**

- Introduce yourself, your institution and the research project.
- Read the research project description aloud. Inform participants that the focus group is expected to take 2 hours. Inform participants of any remuneration provided. Inform participants how their data will be stored. Confirm their right to withdraw their data up until publication of the research results. Provide contact information in case they would like to withdraw their data from the study at a later date.
- Provide an overview of the focus group activities.
- Gather informed consent with a signature for participants who can write or obtain verbal consent with a voice recorder.
- Collect sociodemographic information for each participant (age, gender, etc.).
- Complete Parts 1, 2, and 3.
- Thank participants for joining the focus group, sharing their data and participating in the research.
- Disburse remuneration and request signatures on the participant log to confirm receipt of remuneration.

## Part 1. Food Environment Preference Ranking Activity

[Distribute data collection sheets, beans and pens].

<u>Facilitator Script</u>

Facilitator: Some of you joined the previous focus group on mapping food environments in the community. Today we will continue this theme, but with three different activities that should take approximately 2 hours. Are there any questions about the activity before we start?

[Time for questions].

Facilitator: You have each received a piece of paper. On this paper, you can see different types of food environments along the top of the paper. For new participants who did not participate in the previous focus group, food environments are where we acquire food from. This can be from markets, nature and our relatives and community. Some of you may remember meeting last year to discuss food environments. These places along the top of the paper are where you told me you went to get food last year. Have these places changed or is this still where you go to get food?

Prompt: Is there anywhere else that you acquire food from?

[Time for participants' responses].

Facilitator: Along this side, you can see different characteristics of the places where you go to get food: availability, affordability, access, convenience, and desirability. Let's take some time to explain each of these terms before starting our activity.

Facilitator: Let's start with availability. Does everyone understand what availability means?

[Time for participants' replies].

Availability simply means whether foods are present in that place or not. If you go there will the food item be present? Is the presence of food items in that food environment consistent and reliable? For instance, availability might change by season. Can you think of any examples of how availability changes in the places where you go to get food?

[Time for discussion].

Facilitator: Let us talk for a little bit about convenience. Convenience means how easy and quick something is. If something requires very little time and effort, we will call it very convenient. A place that takes a lot of time and effort to go to, we would call it inconvenient. Does this make sense? Is how we are using the word 'convenient' clear to everyone? Are there any questions about convenience?

[Time for questions].

Facilitator: With access, we are referring specifically to how easily you can travel somewhere. With access, we are thinking about how easy it is to get to a place. For instance, if it is very difficult to go somewhere, we would say it is inaccessible or poorly accessible. If it is very easy to go somewhere, we would say that place has very good access. For instance, last year many of you told me your gardens are very easy to get to. You simply walk out of your door and have already arrived. If this is the case, we would place 5 beans or write the number 5 in the home garden and access square [place 5 beans on that square to demonstrate]. Are there any questions about access?

[Time for questions].

Facilitator: Now, let us talk about desirability. Desirability refers to many things, but mostly it means how much you like something. You might like a place to get food for different reasons. Maybe you like the flavor of the foods there. Maybe you think the foods there are safer or fresher or more delicious. Maybe the foods in that place are important for your culture. Maybe you have positive memories and feelings when you go to that place. Maybe it is fun to go there, either by yourself or with your friends. Can you think of any reasons why you might simply like one place to get food more than another?

[Time for quick discussion].

Facilitator: Does everyone feel like they understand availability, affordability, access, convenience and desirability?

[Time for replies and clarifying discussion if needed].

Facilitator: Now that everyone understands clearly, let us move on to an activity using the sheet of paper in front of you and this bowl of beans. For places where you think the characteristic of that food environment is very good, you can place 5 beans in that square or write the number 5. For instance, if you think the forest is very affordable, you can place 5 beans in the square for forest and affordability or write the number 5. [Place an example of 5 beans on the forest and affordability square]. Where you think the characteristic of the food environment is good, you can place 4 beans or write the number 4. If you think the food environment characteristic in that place is average, you can place 3 beans or write the number 3. If you think that characteristic of the food environment is not good, you can place 2 beans or write the number 2. If you think that characteristic of the food environment is very bad or not good at all, you can place 1 bean or write the number 1. For instance, if you think a place is very expensive or very difficult to get to, you can place 1 bean or write the number 1 in the affordability and access squares. [Place a bean in the affordability + a food environment square to demonstrate]. Does anyone have any questions?

[Time for questions].

Facilitator: I will ask each of you to now place beans or write the number for every square on your sheet. Remember that 5 beans or the number 5 means that that characteristic in that food environment is very good. One bean or the number 1 means that the characteristic for that food environment is not good at all.

[Provide sufficient time for the activity until you see people have put down their pens or finished placing their beans].

Facilitator: Has everyone finished placing their beans or writing the numbers?

[Participants' responses. If participants are finished, take photos of all data sheets].

Facilitator: Now let us come together and discuss the rankings as a group. You can discuss together to decide how many beans to place in each square.

[Time for participants to repeat the activity as a group. Take notes of discussion. Take photo of data sheet once completed].

**Expanded version:**

Facilitator: We will now repeat the activity for the additional characteristics of flavor, satiety, affect, food safety, freshness, healthiness, environmental sustainability and cultural appropriateness.

With flavor, we are referring to how delicious the foods in a food environment are. If foods in that food environment are very delicious, we would rank that food environment with a 5 for taste. If the foods in that food environment taste very bad, then we would give that food environment a rank of 1 for taste. Are there any questions about the taste characteristic?

[Time for questions and answers].

With satiety, we mean how full and nourished you feel after eating food from that food environment. Are there any questions about satiety?

[Time for questions and answers].

With affect, we are referring to how good you feel when you go to that environment. If you feel really good and happy acquiring food from there, then that would be a ranking of 5. If you acquire food from there and feel sad, angry, upset, scared or another negative emotion, that food environment would receive a low ranking for affect. Any questions about affect?

[Time for questions and answers].

Food safety is referring to how safe you think the foods are from that food environment. Do you think foods are safe to eat, or are you worried about contaminants, diseases and hygiene in that food environment? What else could make foods unsafe to eat? Does everyone understand this idea of food safety?

[Time for questions and answers].

With freshness, we are referring to how long foods are stored in that environment. If foods are not stored there, and can be consumed immediately, then we would say those foods are very fresh, and give that food environment a rank of 5 for freshness. If the foods are stored for a long time and are not fresh at all, then we would give that food environment a rank of 1 for freshness. Are there any questions about freshness?

[Time for questions and answers].

Healthiness refers to how healthy you think foods are in a food environment. If you think the foods there are very healthy, you could rank that food environment with a 5. If you think they are average, you could rank that food environment with a 3. If you think the foods there are not really healthy, you can put a ranking of 2. If you think the foods are not healthy at all, you could rank that food environment with a 1. What do you think makes a food environment healthy?

[Time for questions and answers].

Next, we have environmental sustainability. Environmental sustainability refers to how good for the environment acquiring foods from that food environment is. For instance, how good for the environment is foraging, farming or shopping. Good for the environment could refer to the carbon footprint (how much carbon is emitted) or ecological footprint (how much land and how many resources are needed). What other ways do local food environments impact the environment? Are those impacts positive or negative?

[Time for discussion].

Are there any questions about environmental sustainability? Does everyone understand the meaning of this term?

[Time for questions and answers].

Last but certainly not least, we have cultural appropriateness, which refers to whether you can acquire cultural foods from that food environment. What are some of your cultural dishes, and where do you acquire the ingredients?

[Time for discussion].

Are there any questions about cultural appropriateness?

[Time for questions and answers].

Are there any questions about this activity?

[Time for questions and answers].

[Time for activity].

Has everyone finished the ranking the food environments for each characteristic?

Facilitator: We now have one final part of this activity. In this column labelled 'weights,' we will ask you to rank how important you think that characteristic is. For instance, if a characteristic is really important, you would place 5 beans or write the number 5 for that characteristic's weight. If you think that characteristic is not important at all, then you rank it as a 1. If you feel neutral about a characteristic and do not think it is important or unimportant, you can rank it as 'average' with a 3. Are there any questions about this final part of the activity?

[Time for questions and answers].

[Time for weighting part of preference ranking activity].

Has everyone finished assigning weights and ranking the importance of different characteristics?

[Collect data sheets].

**Post-ranking discussion:**

Facilitator: What do you think were the most important characteristics of food environments? Why were those more important than others?

[Time for discussion].

Facilitator: Which characteristics were less important? Why?

[Time for discussion].

Facilitator: Which places do you go to most frequently to get food? Why? Is it because of availability, affordability, accessibility, convenience or desirability?

[Time for discussion].

Facilitator: Which places do you go to rarely for food? Why?

[Time for discussion].

Facilitator: Has where you go to get food and the frequency you go there changed over time? Why? Is it because the availability, access, affordability, convenience or desirability of that place has changed?

[Time for discussion].

Facilitator: I would like to discuss convenience in a bit more detail. Are there times of the year when you have less time? Which times of year? Which places do you go to more frequently during those times of year? What kinds of foods do you eat then?

[Time for participants to respond and discuss].

Facilitator: Let us take a 15-minute bathroom and fruit break.

[Break time. Distribute taste preference ranking data sheets when participants return].

Facilitator: Now let us discuss desirability in more detail. What makes you like a place more? What is important to you?

[Time for participants to respond and discuss].

Facilitator: Would you like a break?

[Optional break depending on participants' energy levels].

## Part 2. Taste Preference Ranking Activity

[Distribute taste preference ranking data collection sheets].

Facilitator: Different food environments have different foods with different flavors. We now have another short activity about your taste preferences. You each have received another data sheet for this new activity. A different flavor is listed in each square: salty, bland, sour, spicy, bitter, sweet, fatty and MSG. You can place 5 beans or write the number 5 in the squares with the flavors that you like the most. For flavors that you like, you can place 4 beans or write the number 4. For flavors that you neither like nor dislike and feel neutral about, you can place 3 beans or write the number 3. For flavors that you dislike, you can place 2 beans or write the number 2. If you do not like that flavor at all, you can place just 1 bean or write the number 1. Are there any questions?

[Time for questions and answers].

[Time for activity].

Facilitator: Has everyone finished?

[If finished, take photos of all data sheets].

**Post-ranking discussion:**

Facilitator: Did anyone notice anything about their data sheet? Did your taste preferences differ from other people? In what ways?

[Time to discuss].

Facilitator: What do you think were the most preferred flavors? Why do you think that is?

[Time to discuss].

Facilitator: What was the least preferred flavor? Why do you think that flavor is less preferred?

[Time to discuss].

Facilitator: Were there any age differences in taste preferences? If so, what were they?

[Time to discuss].

Facilitator: Which places do you associate with specific flavors? For instance, which places do you associate with sweet and bitter foods? Where do you go for salty, spicy, sour or fatty foods?

[Time to discuss].

Facilitator: Do you associate any flavors with positive or negative memories or emotions? Are these emotions and memories related to any specific types of places?

[Time to discuss].

Facilitator: What kind of textures do you like, and where can you get those textured foods?

[Time to discuss].

Facilitator: Are there any places you avoid, because you do not like the flavor there?

[Time to discuss].

Facilitator: Do you avoid getting food from any places for any other reasons?

[Time to discuss].

Facilitator: What are some cultural foods? What kinds of flavors do they have? Where can you get those foods?

[Time to discuss].

Facilitator: How do your cultural foods and food environments compare with those of other social groups? How are the foods different? How are the flavors different?

[Time to discuss].

Facilitator: Would you like a break?

[Optional break depending on participants' energy levels].

**Part 3. Participatory Mapping Activity**

[Distribute printouts of Google Map or Google Earth satellite images and street layouts of areas identified as local food environments, marker pens and white-out[1]].

Facilitator: For the next activity, we will draw where you acquire foods on these maps and images. Can you recognize your home and village in any of these images? Can you recognize any streams, rivers, gardens, fields or forests where you acquire foods? Can you locate any shops, markets, kiosks where you acquire foods?

[Time for inspecting maps and images and discussion].

Can you mark on the map where you acquire foods, and write which foods you acquire there in the margins on the page? You can connect the list of foods to the food environment with a line or arrow.

Does everyone understand the activity? Are there any questions?

[Time for questions and answers].

[Time for activity].

Has everyone finished? Are there any other foods and food environments you use?

[Time for responses. Provide more time if needed].

Which places do you like to go to the most? Why? And, which ones do you like the least? Why?

[Time to discuss].

---

[1] White-out is especially helpful for marking food acquisition locations on darker satellite images of forests.

Elders, are there any foods or food environments that the younger generation does not know about?

[Time to discuss].

[If yes] Why do you think the younger generation does not know about the foods in these places?

Facilitator: Is there anything you would like to tell me or share with me?

[Time to discuss].

Facilitator: Did any activities or questions today bother you? Is there anything you would have done differently or think could have been done better?

[Time for answers].

Facilitator: I would like to thank you all for your time and participation. Thank you for sharing your data with our research project. If you change your mind and no longer want to share your data, remember you can withdraw your data at any time up until we publish the research results. If you decide to withdraw your data, you can still keep the compensation. You received the contact information at the start of the focus group. You can also contact the village leader or the local project contact with any questions or concerns. We hope you enjoyed today's focus group. Thank you all very much for participating today!

## Appendix

Data Collection Sheets:

1.) Simplified food environment preference ranking data collection sheets (5 criteria; no weights) – ENG
2.) Simplified food environment preference ranking data collection sheets (5 criteria; no weights) – THAI
3.) Expanded food environment preference ranking data collection sheets (13 criteria; weights) – ENG
4.) Expanded food environment preference ranking data collection sheets (13 criteria; weights) – THAI
5.) Taste preference data collection sheets – bilingual ENG + THAI

| | Forest | Rotational Farms | Waterways | Home Gardens | Village Kiosk | Fresh Market | Convenience Stores | Supermarkets |
|---|---|---|---|---|---|---|---|---|
| | 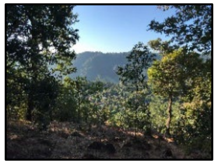 | 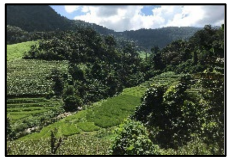 | 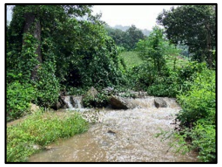 | 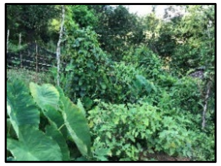 | 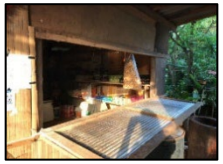 | 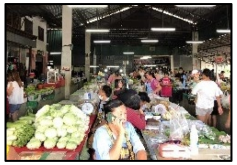 | 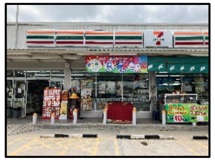 | 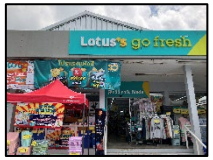  |
| Affordability 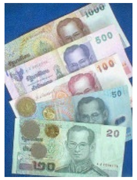 | | | | | | | | |
| Convenience 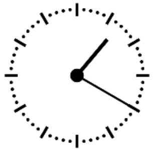 | | | | | | | | |
| Accessibility 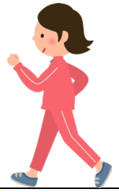 | | | | | | | | |
| Availability 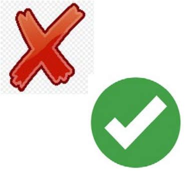 | | | | | | | | |
| Desirability 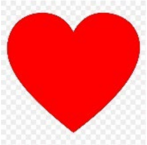 | | | | | | | | |

**1 = Not good at all    2 = Not good    3 = Average    4 = Good    5 = Very good**

| | ป่า | ไร่หมุนเวียน | ตามลำห้วยที่ทำการเกษตร | สวนรอบบ้าน | ร้านขายของชำ | ตลาดสด | เซเว่น | ห้างสรรพสินค้าเช่นโลตัส |
|---|---|---|---|---|---|---|---|---|
| | 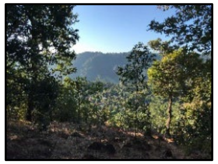 | 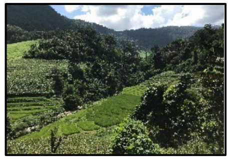 | 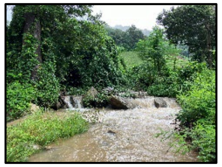 | 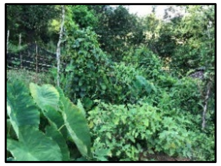 | 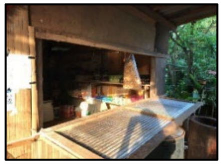 | 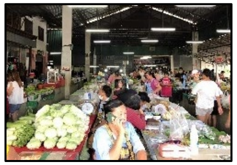 | 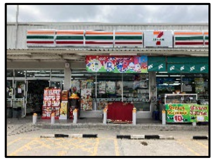 | 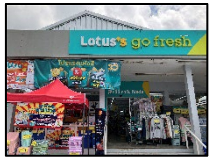  |
| ถูก 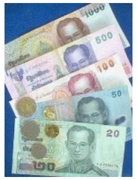 | | | | | | | | |
| สะดวก 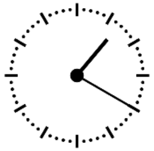 | | | | | | | | |
| ไปง่าย 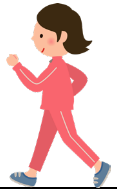 | | | | | | | | |
| มีไหม 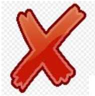 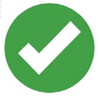 | | | | | | | | |
| รสชาติ สด สะอาด ปลอดภัย วัฒนธรรม 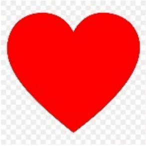 | | | | | | | | |

**1 = ไม่ดีเลย  2 = ไม่ค่อยดี  3 = กลาง ๆ  4 = ดี  5 = ดีมาก**

Date: Participant ID: Age:

| Importance | | Forest 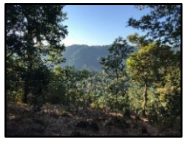 | Rotational Farms 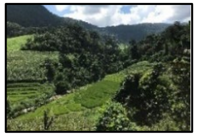 | Waterways 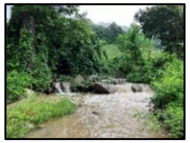 | Home Gardens 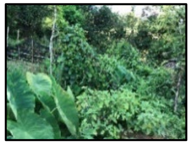 | Village Kiosk 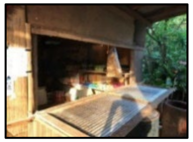 | Fresh Market 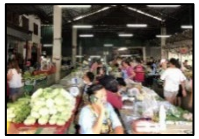 | Convenience Stores 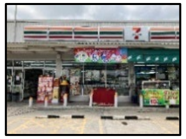 | Supermarkets 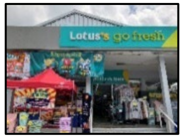 | Kin & Community 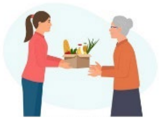 |
|---|---|---|---|---|---|---|---|---|---|---|
| | **Affordability** 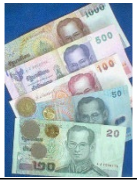 | | | | | | | | | |
| | **Convenience** 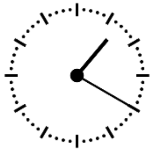 | | | | | | | | | |
| | **Accessibility** 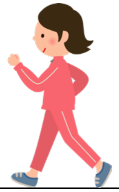 | | | | | | | | | |
| | **Availability** 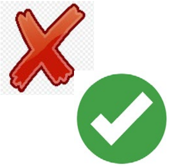 | | | | | | | | | |
| | **Desirability** 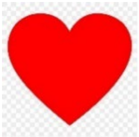 | | | | | | | | | |

**1 = Not good at all 2 = Not good 3 = Average 4 = Good 5 = Very good**

Date: Participant ID: Age:

| Importance | | Forests 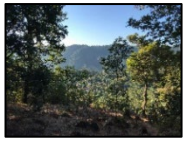 | Rotational farms 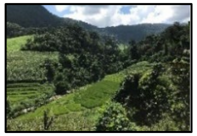 | Waterways 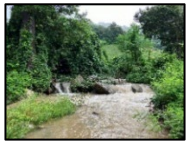 | Home Gardens 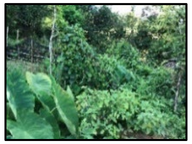 | Village Kiosk 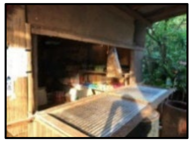 | Fresh Markets 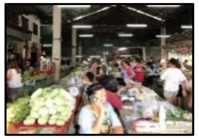 | Convenience Stores 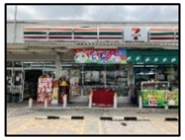 | Supermarkets 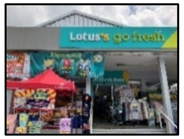 | Kin & Community 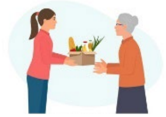 |
|---|---|---|---|---|---|---|---|---|---|---|
| | Taste 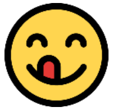 | | | | | | | | | |
| | Satiety 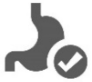 | | | | | | | | | |
| | Affect 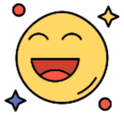 | | | | | | | | | |
| | Food Safety 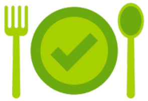 | | | | | | | | | |
| | Freshness 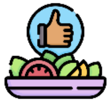 | | | | | | | | | |
| | Healthiness 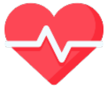 | | | | | | | | | |
| | Sustainability 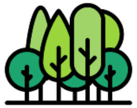 | | | | | | | | | |
| | Culture 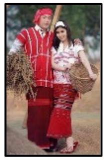 | | | | | | | | | |

**1 = Not good at all 2 = Not good 3 = Average 4 = Good 5 = Very good**

Date วันที่ : Participant ID หมายเลขผู้ร่วม : Age อายุ:

| ความสำคัญ | | ป่า 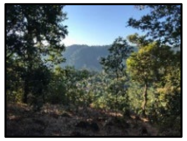 | ไร่หมุนเวียน 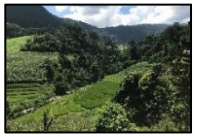 | ลำห้วยที่ทำการเกษตร 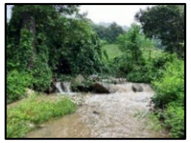 | สวนรอบบ้าน 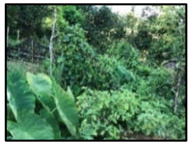 | ร้านขายของชำ 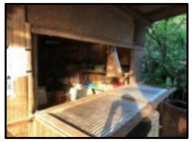 | ตลาดสด 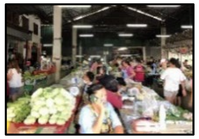 | เซเว่น 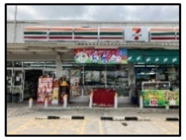 | ห้างสรรพสินค้าเช่นโลตัส 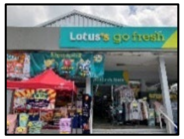 | ชุมชนและพี่น้อง 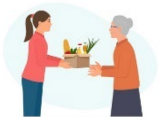 |
|---|---|---|---|---|---|---|---|---|---|---|
| | ถูก 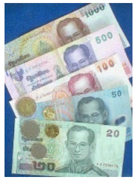 | | | | | | | | | |
| | สะดวก 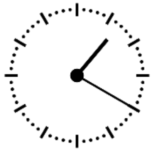 | | | | | | | | | |
| | ไปง่าย 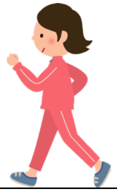 | | | | | | | | | |
| | มีไหม 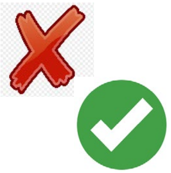 | | | | | | | | | |
| | รสชาติ สด สะอาด ปลอดภัย วัฒนธรรม 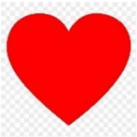 | | | | | | | | | |

**1 = ไม่ดีเลย   2 = ไม่ค่อยดี   3 = กลาง ๆ   4 = ดี   5 = ดีมาก**

Date วันที่ : Participant ID หมายเลขผู้ร่วม : Age อายุ:

| ความสำคัญ | | ป่า 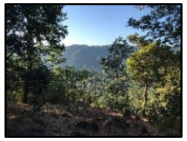 | ไร่หมุนเวียน 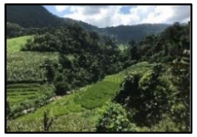 | ลำห้วยที่ทำการเกษตร 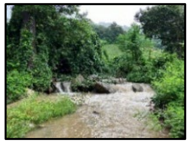 | สวนรอบบ้าน 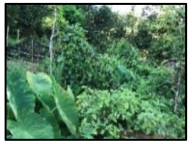 | ร้านขายของชำ 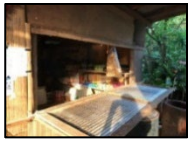 | ตลาดสด 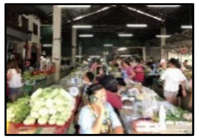 | เซเว่น 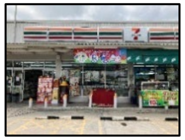 | ห้างสรรพสินค้าเช่นโลตัส 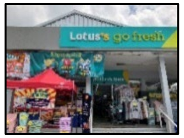 | ชุมชนและพี่น้อง 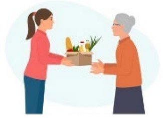 |
|---|---|---|---|---|---|---|---|---|---|---|
| | รสชาติ 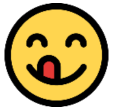 | | | | | | | | | |
| | รู้สึกอิ่ม 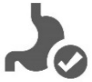 | | | | | | | | | |
| | รู้สึก อารมณ์ดี 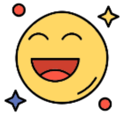 | | | | | | | | | |
| | ปลอดภัย 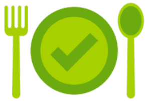 | | | | | | | | | |
| | สด 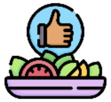 | | | | | | | | | |
| | สุขภาพ 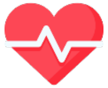 | | | | | | | | | |
| | ธรรมชาติ สิ่งแวดล้อม 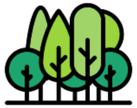 | | | | | | | | | |
| | วัฒนธรรม 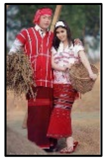 | | | | | | | | | |

**1 = ไม่ดีเลย 2 = ไม่ค่อยดี 3 = กลาง ๆ 4 = ดี 5 = ดีมาก**

| Village: | Participant #: | Age: | Date: |
|---|---|---|---|
| หมู่บ้าน | หมายเลขผู้ร่วม | อายุ | วันที่ |

| | |
|---|---|
| เค็ม Salty 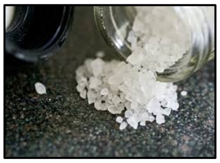 | จืด Bland 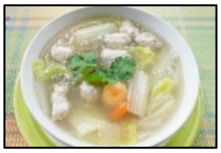 |
| เปรี้ยว Sour 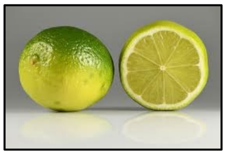 | เผ็ด Spicy 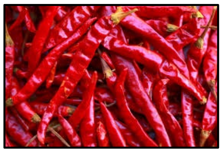 |
| ขม Bitter 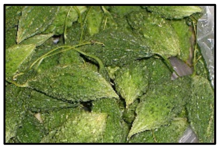 | หวาน Sweet 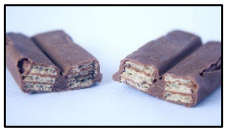 |
| มัน Fatty 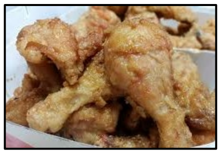 | ผงชูรส MSG 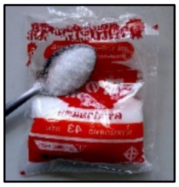 |

1 = ไม่อร่อยเลย Not delicious at all.

2 = ไม่อร่อย Not delicious.

3 = กลาง Average.

4 = อร่อย Delicious.

5 = อร่อยมาก Very delicious.
